# Therapeutic itinerary of pregnant women during COVID-19: a perspective based on healthcare systems*

**DOI:** 10.1590/1518-8345.7623.4629

**Published:** 2025-07-28

**Authors:** Alana Vitória Escritori Cargnin, Camila Moraes Garollo Piran, Beatriz Sousa da Fonseca, Camila Siqueira Floresta Lehmkuhl, Maria de Fátima Garcia Lopes Merino, Adriana Zilly, Marcela Demitto Furtado

**Affiliations:** 1 Universidade Estadual de Maringá, Maringá, PR, Brazil.; 2 Scholarship holder at the Coordenação de Aperfeiçoamento de Pessoal de Nível Superior (CAPES), Brazil.; 3 Universidade Estadual de Maringá, Departamento de Enfermagem, Maringá, PR, Brazil.; 4 Universidade do Oeste do Paraná, Departamento de Enfermagem, Foz do Iguaçu, PR, Brazil.

**Keywords:** COVID-19, Prenatal Care, Pregnancy, Therapeutic Itinerary, Healthcare Models, Women’s Health

## Abstract

to understand the therapeutic itinerary of women who experienced the gestational period during the COVID-19 pandemic.

a descriptive and exploratory study with a qualitative approach, conducted with 16 women who were pregnant during COVID-19. The theoretical framework used was the Health Care Systems Model proposed by Arthur Kleinman. Semi-structured interviews were conducted, which were audio recorded, fully transcribed, and submitted to content analysis as proposed by Bardin and to the IRaMuTeQ software.

the study included women whose ages ranged from 20 to 47 years. Most participants were White and married. Content analysis yielded three categories: 1- Main support networks during pregnancy ― family subsystem; 2- Follow-up during pregnancy ― professional subsystem; and 3- Spirituality as therapeutic support ― cultural subsystem.

it was observed that the paths taken by pregnant women seeking healthcare during the COVID-19 pandemic became even more challenging, being permeated by doubts and uncertainties brought about by the disease, as well as by new configurations of routines and services adopted by healthcare providers.

## Highlights

**(1)** The therapeutic itinerary was influenced by the changes imposed on services. **(2)** Fear of contamination created difficulties in seeking healthcare. **(3)** The healthcare system made it possible to understand the main support networks. **(4)** Spirituality served as support in the face of the difficulties imposed by the pandemic.

## Introduction

On March 11, 2020, a new pandemic was declared by the World Health Organization, caused by the novel coronavirus, named Severe Acute Respiratory Syndrome Coronavirus 2 (SARS-CoV-2), responsible for causing the COVID-19 disease^(1)^. Due to the severity and rapid spread of the disease, risk groups were defined, one of which was the group of pregnant women^(2)^. Although most pregnant women diagnosed with COVID-19 presented with mild to moderate clinical conditions, an increased risk of maternal complications was noted in the presence of associated comorbidities, such as hypertensive disorders and obesity^(3)^.

The risk of maternal mortality was 22 times higher in pregnant women infected with COVID-19 in regions with limited access to healthcare services and where comprehensive Intensive Care Unit (ICU) services were not available^(4)^.

Maternal mortality in pregnant women with COVID-19 may be related to socioeconomic factors, difficulties in accessing healthcare services, and failures in the organization of the system, which were exacerbated by hospital overcrowding. On the other hand, locations with well-structured obstetric care and adequate availability of resources for maternal ICU care presented lower mortality rates^(4)^.

Pregnancy causes several physiological changes in the woman’s body, including modifications in posture, metabolism, and in the cardiovascular, blood, and urinary systems. Changes also occur in cardiac output, the concentration and elevation of certain substances, and adaptations in the respiratory, digestive, and endocrine systems. In the genital organs, transformations are observed in the vulva, vagina, and uterus. Furthermore, there are clinical implications associated with cardiovascular, blood, urinary, respiratory, digestive, and endocrine changes^(5)^.

With the arrival of COVID-19, healthcare services had to reorganize work processes, articulating disease control and prevention strategies and strengthening the entire healthcare network in order to provide users with safety and necessary support^(6)^.

The Ministry of Health recommends a minimum of six consultations throughout prenatal care. However, due to new sanitary measures for virus control, aiming to protect pregnant women from exposure and the risk of contamination in the healthcare unit, in-person consultations became more spaced out than previously recommended^(7)^.

In order to reduce users’ presence in healthcare environments and maintain routine care in health units, teleconsultations combined with in-person visits were a strategy used for prenatal follow-up of pregnant women. Pregnant women who presented symptoms of COVID-19 were also monitored. This measure was constantly evaluated by the team, considering the recommendation protocols of healthcare entities^(8)^.

Even in complex situations, such as a pandemic, it is important to provide access to quality information during the gestational period, for the full experience of this process and autonomy in decision-making related to the pregnancy-puerperal cycle. Moreover, in this context, it is essential to offer individualized care that also values psychosocial aspects. To better understand the experience of these future mothers, it is necessary to identify the paths they followed in seeking care for the resolution of their health problems during the COVID-19 pandemic^(9)^, with this process defined as the Therapeutic Itinerary (TI).

The concept of TI gained relevance when psychiatrist and anthropologist Arthur Kleinman emphasized the cultural influence on the choices made by the ill individual regarding the care pathway, denominating this the theoretical model of Health Care Systems. This model refers to the places where individuals seek solutions for their health problems, classified into three subsystems: family, professional, and cultural^(10-11)^.

In this context, the Therapeutic Itineraries (TIs) represent a research strategy that enables the understanding of the pathways, dynamics, and relationships established by individuals seeking healthcare. Therapeutic Itineraries are configured as a set of interactions and practices constructed by social groups to expand possibilities for access and healthcare assistance^(11)^.

The speed with which the COVID-19 pandemic spread throughout the world was devastating, affecting various populations and age groups, becoming especially severe for the most vulnerable population, such as pregnant women. The difficulties in accessing services and the isolation from their support networks brought pregnant women physical and mental health issues that could affect both mother and fetus. These expose needs and vulnerabilities, as well as exacerbate social, healthcare, and economic inequalities, making it necessary to understand the demands in personal, cultural, and healthcare domains in order to promote comprehensive healthcare that occurs effectively. In light of this, the following question emerged: What was the therapeutic itinerary followed by pregnant women during the COVID-19 pandemic?

In this context, the objective was to understand the therapeutic itinerary of women who experienced the gestational period during the COVID-19 pandemic.

## Method

### Study design

This is a descriptive and exploratory study with a qualitative approach, using the theoretical framework of the Health Care Systems Model proposed by Arthur Kleinman^(10)^.

This theoretical framework addresses the places where individuals seek solutions for their health problems, and it is composed of three subsystems: the family, the professional, and the cultural (the professional consists of formal medical practices, such as biomedicine and homeopathy; the popular includes home care and self-care practices; and the folk refers to mystical and religious health practices)^(10-11)^.

To ensure the quality and transparency of the writing, the Consolidated Criteria for Reporting Qualitative Research (COREQ) guidelines were applied.

### Setting and data collection period

A random draw was conducted, in which ten Primary Health Units (*Unidades Básicas de Saúde* - UBS) were selected for data collection. Data collection occurred between June and October 2023, through semi-structured interviews.

Contact with the participants was established through the provision of data by the health services and direct contact with the women who had scheduled appointments and fit the selected period of the study. Upon consent, the date and time were scheduled according to the participant’s availability, and the interviews were conducted by the nurse researcher, either at the participants’ homes or in spaces provided by the UBS, according to the participant’s preference, always ensuring full privacy.

### Participants and selection criteria

Participants in the study were women who experienced pregnancy during the COVID-19 pandemic, residing in the municipality of Maringá, a city located in the state of Paraná, Brazil, and who belonged to one of the 34 Primary Health Units (UBS, its acronym in Portuguese) in the municipality.

The identification process of these women occurred through the UBS system, by searching for all children born during the pandemic period. Among these, those whose maternal gestational period took place between March 2020 and June 2021 were considered, as this was the period established based on the pandemic in Brazil and the relaxation of restrictive measures against COVID-19 in the country^(12)^.

Inclusion criteria were: women who experienced pregnancy between March 2020 and June 2021, registered at the selected UBS at the time of the interview, and who began prenatal care in the public healthcare system, regardless of their place of residence at the time of pregnancy. Women under 18 years of age and those with any cognitive impairment that would prevent understanding of the interview questions were excluded.

### Instrument and data collection procedures

Before starting each interview, the Informed Consent Form was read aloud, and permission for recording was requested, in addition to providing information about the research and clarifying any doubts.

The interviews were conducted by a nurse researcher with previous experience in scientific research, using the following guiding question: “Tell me what it was like for you to be pregnant during the COVID-19 pandemic.” In addition, supporting questions previously defined by the researcher were used, addressing aspects such as the discovery of pregnancy, restrictive measures imposed on pregnant women, difficulties and/or facilitators encountered in accessing health services, and the healthcare services attended during the pandemic, among other aspects related to pregnancy and the pandemic period. These questions were included to achieve the study objective; a sociodemographic questionnaire was also applied, with questions such as age, race/skin color, number of children, and income, to better understand the participants’ characteristics.

There were no refusals to participate in the study, and the final number of participants was determined during the interviews when theoretical saturation was reached: when the meanings expressed during the interviews became repetitive and no new information emerged, data collection was concluded and the number of participants was established^(13)^.

### Data treatment and analysis

All interviews were recorded and subsequently transcribed in full, with a mean duration of 22 minutes. The material was coded using the IRaMuTeQ software, and the results were processed through thematic analysis as proposed by Bardin^(14)^. This process involved three researchers who conducted the analysis independently. There were no conflicts in the interpretation of the results. The corpus for analysis was compiled after literal transcription, linguistic adaptation, and standardized structuring of the texts, thereby ensuring data reliability.

Content analysis was divided into three stages: 1) familiarization with the material followed by systematization and coding of the data; 2) aggregation of raw data into homogeneous units that facilitate the description and characterization of the content, organizing them into units of meaning; 3) inference based on data previously present in the literature related to the theme, associated with the findings obtained^(15)^.

Subsequently, data triangulation was carried out, combining different analytical strategies to strengthen the validity of the findings. Among these, similarity analysis was employed, based on graph theory, which makes it possible to identify co-occurrences between words and their connections in the textual corpus. This type of analysis supports the understanding the structure of the content, allowing for the visualization of common patterns and specificities in the material, thereby reinforcing the interpretation of the data^(15-16)^.

### Ethical aspects

The research project was approved by the Research Ethics Committee under Authorization No. 4.886.085 (Certificate of Presentation for Ethical Consideration - CAAE: 39060120.1.3004.0104), and all participants signed the Informed Consent Form, in accordance with Resolution No. 466/12 of the National Health Council. To ensure participant anonymity, the names of the interviewees were replaced with the names of flowers.

## Results

A total of 16 women participated in the study, with ages ranging from 20 to 47 years. The majority of participants were white (56.3%), married (68.8%), and multiparous (62.5%), with an income ranging from one to five minimum wages, with most earning between one and two minimum wages (56.3%).

**Figure 1-:**
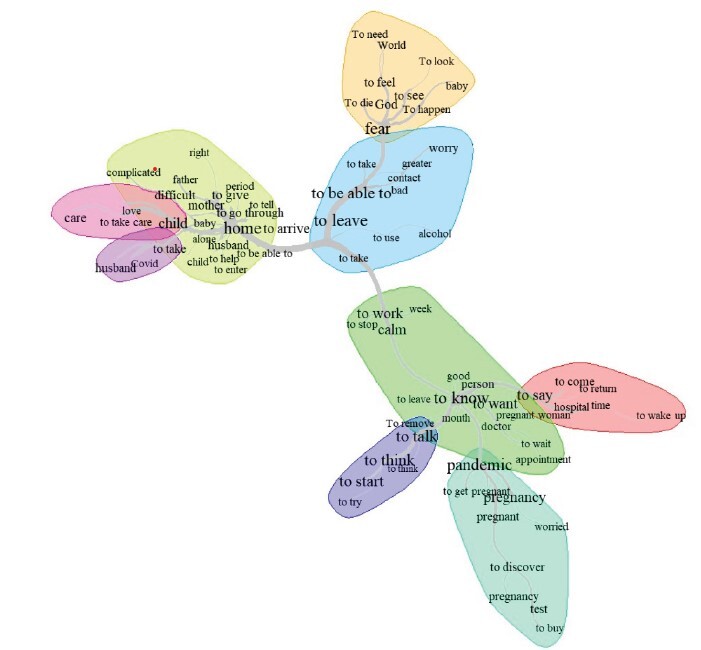
Similarity of words extracted from the summaries of the therapeutic itinerary of pregnant women during the COVID-19 pandemic. Maringá, PR, Brazil, 2023

Through data triangulation, the similarity of words in their structure, central core, and in the peripheral system of the content of the analyzed narratives was observed, with three main organizing axes identified, focusing on the paths taken by pregnant women seeking care during the pandemic period, as shown in Figure 1.

From the extensive reading and data triangulation, it became evident that the experience of women who went through pregnancy during the pandemic was marked by a strenuous journey seeking care, which led to the construction of three categories, described below.

### Main support networks during pregnancy – family subsystem

The word “to be” is closely related to pregnancy, with “be pregnant” revealing that upon discovering the pregnancy, the women sought out their family members or close friends acquaintances. Therefore, they initially sought care when they shared news of the pregnancy with someone, in the expectation, whether conscious or not, of receiving affection, support, comfort, and companionship throughout the pregnancy during a period of great uncertainty, such as the COVID-19 pandemic.

*My husband was with me when I took the test, so we found out together.* (Tulip). *I spoke to my mother first, and then she advised me to go to the health center.* (Azalea). *The first person was my niece, I called her crying [...].* (Violet).

The presence of the family during pregnancy was hindered by restrictions imposed to reduce transmission, whether during medical appointments or due to a perceived decrease in the support received throughout the pregnancy. The experiences of pregnant women in seeking healthcare services alongside family members were affected by the adversities brought on by the COVID-19 pandemic.

*My mother only arrived when I went to have the baby. Without the pandemic, my sisters and my mother could have come, but everything was shut down, no one could travel. So, I think without the pandemic I would have had more support.* (Lily). *My family wanted to accompany me and could not do so very much, so I had to choose between my husband or another family member.* (Gardenia). *My whole family is very participative in some respects. Very welcoming. But, because of the pandemic, we had to distance ourselves.* (Daisy).

In addition to the emotional support provided by family, during the pandemic pregnant women also needed financial support due to the restrictions imposed, which altered their work routines, with many women being removed from their professional duties due to being in the COVID-19 risk group.

*My aunts helped me with everything, with food, I had a lot of support, even some financial support.* (Rose). *We went through a terrible financial period, there was no financial support. I had no income and he was unemployed [...] I asked for a food basket because I had to raise small children.* (Hydrangea). *Pregnant women could not work, and that affected me a lot.* (Daisy).

The women also sought out friends or people they felt close to, either due to the absence of family or because they felt more comfortable in the presence of their friends.

*So, I first told a friend of mine, we talked a lot and cried together. I do not have many relatives here, but I had full support from my friends.* (Orchid). *My teachers helped me a lot as well. I had one teacher with whom I clarified a lot of doubts, she helped me a great deal.* (Gardenia).

The presence of family or a close friend during pregnancy makes the experience lighter and more manageable, and the support may occur both emotionally and financially. Women wish to feel safer and more comfortable in the presence of someone from their usual social circle during the gestational period.

### Prenatal care – professional subsystem

The words “go” and “to do” convey seeking healthcare services essential for a healthy pregnancy. Considering this, it is recommended that prenatal care begin as early as possible to enable comprehensive maternal-fetal monitoring and assessment, with a minimum of six consultations, addressing all needs that may arise during pregnancy.

*I went with my current husband, then I came to this same health center and I already started prenatal care. I was taking medication for depression, and I was referred to high-risk care.* (Rose). *It took me a few days to manage to come here and start the prenatal care. Also, I had had a miscarriage in my first pregnancy, so I preferred to have an ultrasound first to be sure there was a baby, and only then come here and begin prenatal care. I did the entire prenatal care at the health center.* (Anthurium).

Good prenatal care fosters a bond between the healthcare team and the pregnant woman, enabling a good relationship and facilitating the care process. Such closeness benefits not only the pregnancy but also the puerperium, during which healthcare needs remain prominent.

*I received more special care because it was a high-risk pregnancy. So I was going to two healthcare units. The health center and another unit whose name I do not remember. But they treated me very well there and examined me. I had a lot of care there.* (Petunia). *I was very well treated, very well prepared, I had support from a psychiatrist, psychologist, [...] when I came for prenatal care they already referred me, and then it went really smoothly. I had support from both, the psychiatrist and the psychologist.* (Orchid).

In a delicate moment such as the COVID-19 pandemic, healthcare services had to adapt to meet emerging needs; among these was the shortage of medical consultations.

*I only had my first consultation with them, I would only go through triage, there were no doctors to see us because of COVID [...] they scheduled the consultations, but then when you arrived at the health center, the doctors were at the emergency care unit. Because of COVID. Because there was high demand and so they had to leave the health center to go attend patients there.* (Gerbera). *During the pandemic, there were not that many consultations available, a lot of people needed care, so it was hard to get an appointment. And to go there you always had to be careful, because there were people coming in for prenatal care, but there were also other appointments, so we did not know the risk we were facing.* (Azalea).

In addition to the reduction in the availability of medical consultations, the availability of exams was also affected. Difficulty accessing exams during pregnancy is a problem for monitoring gestational development and puts the dyad at risk. Moreover, some exams must be carried out in accordance with gestational age, and the absence of such exams affects the pursuit of care, altering the paths taken by pregnant women seeking healthcare.

*I started prenatal care and also started taking the exams, but I could not get the exams at the health center, so I ended up doing them privately.* (Azalea). *I had an ultrasound done privately. Waiting through the public system took longer. And I had the prenatal care at the health center.* (Lavender).

The changes imposed by the COVID-19 pandemic altered the flow of prenatal care, from the simplest procedures, such as vaccinations, to the most complex, such as childbirth. The pursuit of care was altered, influenced by the availability of healthcare services to attend to pregnant women, which was experienced differently by each woman.

*I was able to get the vaccines through the public system smoothly, but not with the same promptness as in normal times. [...] with the public system you have to schedule, and there is a demand by pregnant women. On the day of the delivery, in my case, I would be seen by the on-call physician. So I opted for private insurance, since I could choose the professional who would accompany me.* (Sunflower). *In my first pregnancy I did everything through the private system, but if I were to have another child and had the option to choose, I would choose the public system. I was much better treated here. Even during the pandemic.* (Begonia).

Another relevant factor was the infection of pregnant women and seeking medical care.

*At seven months I caught COVID. We were really scared, afraid something might happen to the baby, that I would have more severe symptoms, what medications I could take, because being pregnant, I was limited in the medications I could take. But then I had my obstetric consultation, the doctor recommended some more symptomatic medications.* (Gardenia).

A pregnant woman infected with SARS-CoV-2 may present symptoms ranging from mild flu-like symptoms, such as fever and cough, to more severe symptoms, such as respiratory issues, requiring specialized medical care. Moreover, the pandemic and the fear of contagion caused psychological repercussions due to the restrictions and uncertainties imposed on these women. Therefore, the therapeutic itinerary of women who contracted COVID-19 during pregnancy varied depending on the severity of the disease and the availability of healthcare services, since at that time there was often a predetermined path.

*I was 37 weeks along, I was hospitalized for a short time at the maternity ward. To check the baby’s heart rate because of COVID-19. Everything was fine. They sent me home. I took medication at home.* (Petunia). *It was a healthy pregnancy until 35 weeks [...] on Wednesday I took a test. And it came back positive. I already felt some shortness of breath. But I thought it was normal. [...] I had a heart attack, two hospital-acquired bacterial infections, I spent 45 days intubated, 4 and a half months with a tracheostomy, 95% of my lungs were affected, and I was in the hospital for 53 days*. (Magnolia).

It is clear that the difficulties imposed by the COVID-19 pandemic reduced access to healthcare services, introducing many vulnerabilities to the provision of quality prenatal care, as women attended fewer consultations and exams. Consequently, they had fewer opportunities for health education and early detection of potential pregnancy-related problems.

### Spirituality as therapeutic support – cultural subsystem

It was observed that pregnant women turned to their spiritual beliefs during this pandemic period, which served as therapeutic support to help them feel strengthened in the face of infection and the adversities imposed by COVID-19.

*I always put my health in God’s hands. So for me, I always remained strong in God’s presence. However, I caught COVID, and I was near death. But God was there, He comforted me, healed me.* (Petunia). *That is what we did at home, we prayed, and we were prepared for anything, because many people died, right, many people died, so we did not know what was coming.* (Hydrangea).

The women’s spirituality played an important role in giving them encouragement and courage to seek prenatal care services in the midst of the pandemic. Religiosity allowed them to believe that the pregnancy would unfold healthily, even during the pandemic.

*Faith in God for protection and out of necessity, because I knew I needed it, [...] I needed professional help. (Magnolia). It was something I thought would never happen again, so it was a good surprise. I said: it was not planned by me, but it was planned by God, because He knew I needed Him.* (Orchid).

It is evident that the pregnancy was related to a greater plan, in which the pandemic had an influence and maternal desires could not be fulfilled.

*On the day I went to give birth, I was hoping I would be able to record the delivery, it was a dream I had, to be able to film [...] I called the photographer I had hired, we had already agreed that if the hospital allowed, he would come in, [...] but the hospital denied his entry due to the increase in cases, [...] I was not able to fulfill this wish. I really wanted to film the entire birth, to have professional photos to keep forever. So the pandemic threw everything off, and I was not able to fulfill some of the wishes I had, I had to stay away from many places I would have liked to go during my pregnancy.* (Daisy).

The experiences along the path taken were multiple, revealing both positive and negative aspects, depending on each pregnant woman’s personal experience.

## Discussion

This study enabled an understanding of the effects of the COVID-19 pandemic on the dynamics of seeking care during the gestational period. The findings indicate that COVID-19 led to changes in the dynamics of healthcare services, as well as in the support network available to pregnant women, affecting changes in the TI experienced by them.

Despite so many adversities, it was possible to observe the women’s pursuit of care, both through healthcare services and within the family and cultural spheres, which demonstrates a certain adaptive capacity and resilience.

The pregnant women seeking family support, a fundamental element in the interviews, is linked to the social support network, composed of individuals significant to them who provide support and reinforcement in coping strategies in the face of life situations. This network includes the extended family, friends, coworkers, community relationships, healthcare services, and even religious or political beliefs, encompassing both intimate and occasional relationships^(17-18)^.

From this perspective, Kleinman^(19)^ explains in his theory that Health Care Systems are based on interpersonal relationships, where everything is interconnected. Patients, healthcare providers, illness, and treatment are all part of this care context. Individual beliefs and attitudes are shaped by cultural roles. The author further emphasizes that, in order to understand individual choices, it is essential to consider the external influences that affect the Health Care System, such as environmental factors, including geography, climate, demography, social issues, pollution, and agricultural and industrial development^(19)^.

Therapeutic itineraries refer to the health care each person adopts, shaped by interpersonal relationships. The choices regarding care are influenced by three sectors: the family, which has the greatest power in defining the therapeutic itinerary; culture, which includes professionals not officially recognized but who share the individuals’ social beliefs and cultural values; and healthcare providers, who have a more limited influence, as they tend to prioritize the biological aspect of care^(19)^.

In addition, the theory emphasizes the plurality of care, arguing that individuals move between care sectors (family, cultural, and professional), revealing a combination of different forms of care, reinforcing that the therapeutic itinerary is not linear and may include formal and informal treatments simultaneously^(19)^.

Social support plays a crucial role in preventing postpartum depression, highlighting the importance of paying attention to the quality of the support network for pregnant women during the pregnancy-puerperal cycle. In this process, it is not only the family, but also healthcare services, friends, and partners that assume responsibilities in supporting women during this period^(20)^.

From this perspective, the objective of prenatal care is to ensure the healthy development of pregnancy, childbirth, and the puerperium, without compromising the health of the mother and baby. This involves an approach that also considers psychosocial aspects, in addition to educational and preventive activities. This objective is a responsibility shared between the physician and the nurse^(8)^.

The healthcare provider must develop a healthy bond with the pregnant woman and her family. By encouraging this support network to attend prenatal follow-up consultations, it is understood that these are the moments when doubts are clarified and there is the possibility for everyone to feel more reassured and secure about the gestational process^(21)^.

Nursing assumes responsibility for the health care of these women by developing educational actions based on individuality, integrality, self-care, and female empowerment, enabling them to make their own decisions and contributing to their independence regarding health demands, encouraging them to become jointly responsible for their own care^(22)^.

However, due to the coronavirus pandemic, it was necessary to make adaptations in the care provided to pregnant women, aiming to preserve the health of both this population and the healthcare providers involved. Due to physiological changes in the immune, cardiorespiratory, and coagulation systems, pregnant women at any stage of pregnancy and postpartum women were classified as part of the risk group for COVID-19, with a greater likelihood of worsening the infectious condition, thus requiring more intensive care^(23)^.

Such care is necessary due to the high transmissibility of the disease, which occurs through respiratory droplets expelled when an infected person coughs or sneezes, inhaled by nearby individuals. It can also occur through personal contact, close contact, or contact with contaminated surfaces, followed by touching the mouth, nose, and/or eyes^(1)^.

In response to this situation, health authorities implemented measures aimed at reducing the spread of the virus, emphasizing the importance of hand hygiene, mask use, reorganizing healthcare service appointments, and adopting social isolation^(24)^.

On the other hand, social isolation, as a way to prevent the spread of COVID-19, may have led to feelings of loneliness, as it distanced the woman from her support network, which assisted with household tasks, guidance, and emotional support^(25)^. A study conducted in 2020 revealed that pregnant women during the COVID-19 pandemic reported higher levels of distress and psychiatric symptoms compared to women evaluated before the pandemic, with progression to cases of depression and anxiety. The authors concluded that the lack of clear and reliable information could exacerbate the risk of psychological and psychosocial distress among these pregnant women^(26)^.

Implementing tools to screen for the risk of depression during pregnancy may be an important step in better understanding the mental health needs of women receiving routine risk prenatal care. It is known that, often, mental health is neglected and, in addition, the difficulty of access to specialized care in this area within primary healthcare must be considered^(27)^.

During the pandemic, innovation and creativity became part of the daily life of the healthcare provider who, on a daily basis, faced new challenges. It was necessary to resort to adaptation strategies to meet the existing health calendars, which at that moment needed to be carried out in some way^(28)^.

Regarding maternal infection by coronavirus, there are reports that pregnant women are more likely to develop preeclampsia, gestational diabetes, and hypertension, and HELLP syndrome^(29)^. Another study demonstrated an association between maternal infection and fetal outcomes, such as preterm birth, fetal distress, low birth weight, asphyxia, premature rupture of membranes, disseminated intravascular coagulation, and even fetal death^(30)^.

The complications arising from pregnancy may lead to hospitalization, which, although promoting a sense of safety and protection, also arouses in the pregnant woman feelings of distress, pain, anger, sadness, and fear resulting from the disruption of routine and the interventions and procedures performed^(31)^.

The professional subsystem, composed of services and healthcare providers, is bureaucratically organized and involves formal learning and training, being legally recognized and attracting society due to its influence and healing capacity^(11)^.

Regarding cultural aspects, religion has a strong influence on quality of life in different life stages, becoming relevant to promoting health and preventing harm in the population^(32)^. The spiritual dimension in health care during pregnancy is a fundamental resource to promote autonomy, security, and comfort, in addition to strengthening the maternal-fetal bond. Spirituality, in this sense, is a phenomenon that gives meaning to each stage of life, while also providing purpose to human existence itself^(33)^.

By seeking both formal and informal care, the distance between these systems is reduced, enabling care practices that are multicultural and meaningful. This allows for a comprehensive health approach that considers cultural care, emphasizing that, in this way, new knowledge may emerge, contributing to the transformation of health practices^(34)^.

The limitations of the study refer to the recall bias of the participants interviewed due to the time elapsed between pregnancy and data collection for the research. However, it is noteworthy that pregnancy is a very significant period for women and, combined with the pandemic, a historical event, likely little or nothing was forgotten.

The findings of this study are extremely relevant, given the numerous changes imposed on healthcare services during the pandemic period, with some remaining after the pandemic, such as teleconsultations.

Moreover, the results may contribute to the professional practice of those who provide prenatal care to pregnant women, improving their understanding of the paths taken seeking care, as well as the need for inclusion and acceptance of family members and cultural aspects throughout the prenatal follow-up.

## Conclusion

The therapeutic itinerary followed by pregnant women seeking healthcare during the COVID-19 pandemic was shaped by three subsystems: familial, professional, and cultural. Women first sought support from their families, seeking affection, care, guidance, and assistance, and then turned to professional support, primarily through Primary Health Care, which served as the main entry point for care throughout pregnancy, childbirth, and the postpartum period. Many women also engaged with cultural beliefs as a tool for strengthening their care, in combination with the other subsystems.

Experiencing pregnancy during the COVID-19 pandemic made the pathways to seeking care even more difficult for these women, being permeated with doubts and uncertainties, as well as a new configuration of routines and appointments adopted by healthcare services.

Many pregnant women faced dilemmas about seeking healthcare services during the pandemic, highlighting the urgent need to ensure continuous access to prenatal care. It is believed that this study may provoke reflection on the importance of individualized and holistic care for pregnant women, aiming to understand and respect their beliefs and ways of life, their understanding, anxieties, and immediate needs, in order to improve prenatal care, especially in adverse situations, such as the COVID-19 pandemic.
