# Supplementary material for: Therapeutic itinerary of pregnant women during COVID-19: a perspective based on healthcare systems
Source: Rev Lat Am Enfermagem. 2025 Jul 28;33:e4629. doi: 10.1590/1518-8345.7623.4629 (PMC12306935; doi:10.1590/1518-8345.7623.4629)
Supplement: Translation

# Translation: Itinerário terapêutico de gestantes durante a COVID-19: um olhar a partir dos sistemas de cuidado*

**DOI:** 10.1590/1518-8345.7623.4630

**Authors:** Alana Vitória Escritori Cargnin, Camila Moraes Garollo Piran, Beatriz Sousa da Fonseca, Camila Siqueira Floresta Lehmkuhl, Maria de Fátima Garcia Lopes Merino, Adriana Zilly, Marcela Demitto Furtado

**Affiliations:** 1 Universidade Estadual de Maringá, Maringá, PR, Brasil.; 2 Bolsista da Coordenação de Aperfeiçoamento de Pessoal de Nível Superior (CAPES), Brasil.; 3 Universidade Estadual de Maringá, Departamento de Enfermagem, Maringá, PR, Brasil.; 4 Universidade do Oeste do Paraná, Departamento de Enfermagem, Foz do Iguaçu, PR, Brasil.

**Keywords:** COVID-19, Cuidado Pré-Natal, Gestação, Itinerário Terapêutico, Modelos de Assistência à Saúde, Saúde da Mulher

## Abstract

compreender o itinerário terapêutico de mulheres que vivenciaram o período gravídico durante a pandemia da COVID-19.

estudo descritivo e exploratório com abordagem qualitativa, desenvolvido com 16 mulheres que gestaram durante a COVID-19. Utilizou-se como referencial teórico o Modelo de Sistemas de Cuidado à Saúde, proposto por Arthur Kleinman. Foram realizadas entrevistas semiestruturadas, as quais foram audiogravadas, transcritas na íntegra e submetidas à análise de conteúdo proposta por Bardin e ao software IRaMuTeQ.

participaram do estudo mulheres com idades que variaram de 20 a 47 anos. A maioria das participantes era da raça/cor branca e casada. A partir da análise de conteúdo, emergiram três categorias: 1 - Principais redes de apoio durante a gestação ― subsistema familiar; 2 - O acompanhamento durante a gestação ― subsistema profissional; e 3 - A espiritualidade como suporte terapêutico ― subsistema cultural.

notou-se que os caminhos percorridos por gestantes em busca do cuidado em saúde, durante a pandemia da COVID-19, tornaram-se ainda mais difíceis, estando permeados por dúvidas e incertezas trazidas pela doença, bem como pelas novas configurações de rotinas e atendimentos adotados pelos serviços de saúde.

## Destaques

**(1)** O itinerário terapêutico foi influenciado diante das mudanças impostas aos serviços. **(2)** O medo da contaminação impôs dificuldades na busca pelos cuidados em saúde. **(3)** O sistema de cuidados à saúde possibilitou entender as principais redes de apoio. **(4)** A espiritualidade foi suporte diante das dificuldades impostas pela pandemia.

## Introdução

Em 11 de março de 2020, foi decretada pela Organização Mundial da Saúde uma nova pandemia, causada pelo novo coronavírus, denominado *Severe Acute Respiratory Syndrome Coronavírus* 2 (SARS-CoV-2), responsável por causar a doença COVID-19^(1)^. Devido à gravidade e à rápida disseminação da doença, foram definidos grupos de risco, sendo um deles o grupo das gestantes^(2)^. Embora a maioria das gestantes diagnosticadas com COVID-19 tenha apresentado quadros clínicos leves a moderados, nota-se aumento do risco de complicações maternas diante da presença de comorbidades associadas, como distúrbios hipertensivos e obesidade^(3)^.

O risco de mortalidade materna é 22 vezes maior em gestantes infectadas pela COVID-19, em regiões com menor acesso a serviços de saúde e quando os serviços integrais de Unidade de Terapia Intensiva (UTI) não estão disponíveis^(4)^.

A mortalidade materna em gestantes com COVID-19 pode estar relacionada a fatores socioeconômicos, dificuldades no acesso aos serviços de saúde e falhas na organização do sistema, agravadas pela superlotação hospitalar. Por outro lado, locais com assistência obstétrica bem estruturada e disponibilidade adequada de recursos para UTI materna apresentam índices mais baixos de mortalidade^(4)^.

A gestação provoca diversas alterações fisiológicas no corpo da mulher, incluindo modificações na postura, metabolismo e nos sistemas cardiovascular, sanguíneo e urinário. Também ocorrem mudanças no débito cardíaco, na concentração e elevação de certas substâncias, além de adaptações nos sistemas respiratório, digestório e endócrino. Nos órgãos genitais, transformações são observadas na vulva, vagina e útero. Além disso, há implicações clínicas associadas às alterações cardiovasculares, sanguíneas, urinárias, respiratórias, digestivas e endócrinas^(5)^.

Com a chegada da COVID-19, os serviços de saúde necessitaram reorganizar o processo de trabalho, articulando estratégias de controle, prevenção da doença e fortalecimento de toda a rede de saúde, a fim de proporcionar ao usuário segurança e o apoio necessário^(6)^.

O Ministério da Saúde (MS) recomenda a realização de no mínimo seis consultas ao longo do pré-natal. No entanto, a partir das novas medidas sanitárias para controle do vírus, visando preservar a gestante da exposição e risco de contaminação na unidade de saúde, as consultas presenciais passaram a ter um espaçamento maior que o preconizado anteriormente^(7)^.

Visando diminuir a circulação dos usuários nos ambientes e dar continuidade ao cuidado rotineiro nas unidades de saúde, os atendimentos de teleconsulta associados aos presenciais foram uma estratégia utilizada no acompanhamento das gestantes no pré-natal. As gestantes que apresentavam sintomas da COVID-19 também eram monitoradas. Esta medida foi avaliada constantemente pela equipe, considerando os protocolos de recomendações das entidades de saúde^(8)^.

Mesmo em situações complexas, como em uma pandemia, torna-se importante proporcionar acesso à informação de qualidade durante o período gestacional, para a plena vivência desse processo e autonomia diante das escolhas que envolvem o ciclo gravídico-puerperal. Além disso, neste contexto, é indispensável oferecer uma assistência individualizada e que valorize, também, seus aspectos psicossociais. E, para melhor compreensão da experiência dessas futuras mães, torna-se necessário saber os caminhos percorridos por elas na busca de cuidados para resolução dos seus problemas de saúde durante a COVID-19^(9)^, sendo este processo definido como Itinerário Terapêutico (IT).

O conceito de IT ganhou importância quando o psiquiatra e antropólogo Arthur Kleinman deu ênfase à influência cultural nas escolhas do doente relativas ao percurso de cuidado, sendo este denominado modelo teórico dos Sistemas de Cuidados à Saúde. Este modelo refere-se aos locais onde os indivíduos buscam soluções para seus problemas de saúde, classificados em três subsistemas: familiar, profissional e cultural^(10-11)^.

Nesse contexto, os Itinerários Terapêuticos (ITs) representam uma estratégia de pesquisa que permite compreender os percursos, dinâmicas e relações estabelecidas pelas pessoas em sua busca por cuidados em saúde. Os ITs configuram-se como um conjunto de interações e práticas construídas pelos grupos sociais para ampliar as possibilidades de acesso e assistência à saúde^(11)^.

A velocidade com que a pandemia da COVID-19 se disseminou pelo mundo foi devastadora, atingindo diversos públicos e faixas etárias, tornando-se grave especialmente para a população mais vulnerável, como as gestantes. As dificuldades de acesso aos serviços e o isolamento de seus núcleos de apoio trazem às gestantes dificuldades de saúde físicas e mentais que podem afetar a ela e ao feto. Expõem as necessidades e fragilidades, como também exacerbam desigualdades sociais, sanitárias e econômicas, tornando-se necessário compreender as demandas nos âmbitos pessoais, culturais e de saúde, para que se possa promover uma assistência de saúde integral e que ocorra de forma efetiva. Em face disso, emergiu a seguinte questão: Qual o itinerário terapêutico percorrido por gestantes durante a pandemia da COVID-19?

Neste contexto, o objetivo foi compreender o itinerário terapêutico de mulheres que vivenciaram o período gravídico durante a pandemia da COVID-19.

## Método

### Delineamento do estudo

Estudo descritivo e exploratório com abordagem qualitativa, utilizando o referencial teórico do Modelo de Sistemas de Cuidados à Saúde proposto por Arthur Kleinman^(10)^.

Esse referencial teórico aborda os locais onde os indivíduos buscam soluções para os seus problemas de saúde, e o mesmo é composto por três subsistemas: familiar, profissional e cultural (o profissional, constituído pelas práticas formais de exercício da medicina, como biomedicina e homeopatia; o popular, composto pelos cuidados caseiros e o autocuidado; ou o *folk*, que corresponde a práticas místicas e religiosas de cuidados)^(10-11)^.

Com a finalidade de garantir a qualidade e transparência da redação, foi aplicado o guia *Consolidated Criteria for Reporting Qualitative Research* (COREQ).

### Cenário e período de coleta de dados

Foi realizado um sorteio aleatório, no qual dez Unidades Básicas de Saúde (UBS) foram selecionadas para a coleta de dados. Ela aconteceu entre junho e outubro de 2023, por meio de entrevista semiestruturada.

O contato com as participantes se deu mediante a disponibilização dos dados pelos serviços de saúde e pelo contato direto com as mulheres que tinham consultas agendadas e se encaixavam no período selecionado na pesquisa. A partir do aceite, foram agendados o dia e horário conforme a disponibilidade da participante, e as entrevistas foram realizadas pela enfermeira pesquisadora, nas residências, ou em espaços cedidos pelas UBS conforme a preferência da participante, sempre garantindo sua total privacidade.

### Participantes e critérios de seleção

Participaram do estudo mulheres que gestaram durante a pandemia da COVID-19, residentes no município de Maringá, cidade localizada no interior do Paraná, Brasil, e que pertenciam a uma das 34 UBS do município.

O processo de identificação dessas mulheres aconteceu por meio do sistema das UBS, realizando-se uma busca por todas as crianças nascidas no período pandêmico. Dessas, consideraram-se aquelas nas quais o período gestacional materno tivesse acontecido entre março de 2020 e junho de 2021, sendo este período considerado devido ao início da pandemia no Brasil e ao afrouxamento das medidas restritivas contra a COVID-19 no Brasil^(12),^ respectivamente.

Os critérios de inclusão foram: mulheres que gestaram no período de março de 2020 a junho de 2021, cadastradas nas UBS selecionadas no momento da entrevista e que iniciaram o pré-natal na rede pública, independentemente do local de residência no momento da gestação. Foram excluídas mulheres com idade inferior a 18 anos e aquelas com algum déficit cognitivo que impossibilitasse a compressão das perguntas da entrevista.

### Instrumento e procedimentos para a coleta de dados

Antes de iniciar cada entrevista, efetuou-se a leitura do Termo de Consentimento Livre e Esclarecido (TCLE), solicitando a permissão para a gravação, além de fornecer dados sobre a pesquisa e esclarecer possíveis dúvidas.

As entrevistas foram realizadas por uma enfermeira pesquisadora com experiência prévia na pesquisa científica e, para conduzir as entrevistas, utilizou-se a seguinte questão norteadora: “Conte-me como foi para você gestar durante a pandemia da COVID-19”. Somada a esta, houve questões de apoio previamente delimitadas pela pesquisadora, que estavam relacionadas à descoberta da gestação, às medidas restritivas impostas ás gestantes, às dificuldades/facilidades encontradas no acesso aos serviços de saúde, e com relação aos serviços de saúde frequentados durante a pandemia, entre outros aspectos relacionados à gestação e ao período pandêmico. Essas questões foram inseridas a fim de atingir o objetivo do estudo; também foi aplicado um questionário sociodemográfico, com questões como: idade, raça/cor, quantidade de filhos, e renda para conhecer melhor as características das participantes.

Não houve recusas para a participação do estudo e o número final de participantes foi definido durante as entrevistas em que se chegou à saturação teórica: diante da similaridade dos significados durante as entrevistas e não havendo mais novas informações, a coleta de dados foi interrompida e o número de participantes estabelecido^(13)^.

### Tratamento e análise dos dados

Todas as entrevistas foram gravadas e, posteriormente, transcritas na íntegra, tendo duração média de 22 minutos. O material foi codificado com o auxílio do *software* IRaMuTeQ, sendo os resultados tratados por meio da análise temática proposta por Bardin^(14)^. Neste processo estiveram envolvidas três pesquisadoras que realizaram a análise de forma independente. Não houve conflitos na compreensão dos resultados. O *corpus* para análise foi realizado após a transcrição literal, adaptação linguística e estruturação padronizada dos textos, garantindo assim a confiabilidade dos dados.

A análise de conteúdo foi dividida em três etapas: 1) o reconhecimento de todo o material seguido de sistematização e codificação dos dados; 2) a agregação dos dados brutos em unidades homogêneas que facilitam a descrição e caracterização do conteúdo, organizando-os, então, em unidades de sentido; 3) a inferência sobre dados previamente presentes na literatura a respeito da temática, associada aos resultados encontrados^(15)^.

Posteriormente, foi realizada a triangulação dos dados, combinando diferentes estratégias de análise para reforçar a validade dos achados. Entre elas, utilizou-se a análise de similitude, baseada na teoria dos grafos, que possibilita identificar coocorrências entre palavras e suas conexões no corpus textual. Esse tipo de análise auxilia na compreensão da estrutura do conteúdo, permitindo visualizar padrões comuns e especificidades do material, o que fortalece a interpretação dos dados^(15-16)^.

### Aspectos éticos

O projeto de pesquisa foi aprovado pelo Comitê de Ética em Pesquisa sob o Parecer n.º 4.886.085 (Certificado de Apresentação de Apreciação Ética - CAAE: 39060120.1.3004.0104) e todas as participantes assinaram o Termo de Consentimento Livre e Esclarecido (TCLE), de acordo com a Resolução n.º 466/12 do Conselho Nacional de Saúde. Para assegurar o anonimato das participantes, os nomes das entrevistadas foram substituídos por nomes de flores.

## Resultados

Participaram do estudo 16 mulheres com idades que variaram de 20 a 47 anos. A maioria das participantes era da raça/cor branca (56,3%), casada (68,8%) e multípara (62,5%), com renda que variou entre um e cinco salários-mínimos, sendo que a maioria ganhava entre um e dois salários (56,3%).

Por meio da triangulação dos dados, observou-se a similitude das palavras em sua estrutura, núcleo central e no sistema periférico dos conteúdos das ementas analisadas, sendo identificados três principais eixos organizadores voltados aos caminhos percorridos por gestantes durante a busca por cuidados no período pandêmico, conforme a Figura 1.

**Figura 1-:** Similitude de palavras extraídas das ementas do itinerário terapêutico de gestantes durante a pandemia da COVID-19. Maringá, PR, Brasil, 2023

A partir da leitura extenuante e da triangulação dos dados, percebeu-se que a experiência das mulheres que vivenciaram a gestação durante a pandemia foi marcada por uma trajetória árdua pela busca de cuidado, tendo sido possível a construção de três categorias, descritas a seguir.

### Principais redes de apoio durante a gestação- subsistema familiar

A palavra “estar” se relaciona intimamente à gestação, “estar grávida”, descobrindo-se que, ao perceber a gravidez, as mulheres procuraram seus familiares ou pessoas próximas. Dessa forma, a primeira busca por cuidado se deu ao dividir com alguém a notícia da gestação, na perspectiva, mesmo que inconsciente, de encontrar afeto, apoio, conforto e companhia para trilhar todo o trajeto da gestação num tempo de tamanha incerteza, como foi o da pandemia da COVID-19.

*Meu marido, estava junto comigo quando eu fiz o teste, então descobrimos juntos.* (Tulipa). *Eu falei primeiro com a minha mãe, e aí depois ela me orientou a procurar o posto de saúde.* (Azaleia). *A primeira pessoa foi minha sobrinha, liguei para ela chorando [...].* (Violeta).

A presença familiar durante a gestação foi prejudicada pelas restrições impostas para reduzir a contaminação, seja durante as consultas, seja pela sensação de redução no apoio recebido ao longo da gestação. As experiências das gestantes na procura pelos serviços de saúde junto de seus familiares foram alteradas devido às adversidades trazidas pela pandemia da COVID-19.

*Minha mãe só chegou quando eu fui ganhar o bebê. Sem a pandemia, as minhas irmãs e a minha mãe poderiam ter vindo, mas aí fecharam tudo, não podia viajar. Então, acho que sem a pandemia eu teria tido um apoio maior.* (Lírio). *Minha família queria me acompanhar e não podia muito, aí eu tinha que escolher entre meu marido ou algum outro familiar.* (Gardênia). *A minha família como um todo é bem participativa, em alguns pontos. Bem acolhedora. Mas, por conta da pandemia, a gente precisou se afastar.* (Margarida).

Além do suporte emocional provido pela família, durante a pandemia as gestantes necessitaram de apoio financeiro devido *à*s restrições impostas, as quais modificaram o dia a dia de trabalho, estando muitas gestantes afastadas do exercício profissional por serem grupo de risco para a COVID-19.

*As minhas tias me ajudaram em tudo, em questão de comida, eu tive um apoio bem grande, até um certo apoio financeiro.* (Rosa). *A gente passou um período financeiramente terrível, não tinha um suporte financeiro. Eu estava sem salário e ele estava desempregado [...] pedi cesta básica porque tinha que criar as crianças pequenas.* (Hortênsia). *As gestantes não podiam trabalhar, e isso me abalou muito.* (Margarida).

As mulheres procuraram também amigos, ou pessoas com as quais se sentiam próximas, ou pela ausência de seus familiares ou, ainda, por se sentirem mais confortáveis na presença de seus amigos.

*Então, eu falei primeiro para uma amiga minha, a gente conversou muito e a gente chorou junto.Parentes eu não tenho muito aqui, mas das amizades eu tive apoio total.* (Orquídea). *Meus professores me ajudaram bastante também. Eu tinha uma professora que eu tirava muita dúvida com ela, ela me ajudou muito.* (Gardênia).

A participação familiar, bem como de um amigo próximo, durante a gestação, torna a experiência mais leve e mais tranquila, e o apoio pode acontecer tanto no suporte emocional como financeiro. É perceptível que as mulheres desejam sentir-se mais seguras e confortáveis na presença de um indivíduo de seu convívio habitual, durante o período gestacional.

### O acompanhamento pré-natal – subsistema profissional

As palavras “ir” e “fazer” transmitem a procura pelos serviços de saúde, sendo este essencial para uma gestação saudável. Diante disso, é recomendado o início do pré-natal o mais precocemente possível, para acompanhamento e avaliação materno-fetal, que devem ser completos, sendo realizadas no mínimo seis consultas e suprindo todas as necessidades que possam surgir durante a gestação.

*Eu fui com o meu atual esposo, aí eu já vim para esse mesmo postinho e eu já dei início no pré-natal. Aí eu tomava remédio para a depressão, e eu fui encaminhada pro alto risco.* (Rosa). *Eu demorei uns dias para conseguir vir aqui fazer, dar início a esse pré-natal. Até porque eu tive um aborto na minha primeira gestação, então eu preferi fazer um ultrassom primeiro, para ter certeza que tinha um bebê, para depois vir aqui começar o pré-natal. E aí eu fiz todo o pré-natal pelo posto.* (Antúrio).

O bom atendimento pré-natal gera vínculo entre a equipe de saúde e a gestante, possibilitando um bom relacionamento e facilitando o processo de cuidado. Tal proximidade irá beneficiar não somente a gestação, mas também o período de puerpério, no qual as necessidades de saúde permanecem em evidência.

*Eu tive um cuidado mais especial pelo fato de ter gestação de risco. Então eu fiquei indo em duas unidades de saúde. No postinho e uma outra unidade que eu não me lembro o nome. Só que eles me atendiam muito bem lá e eles me examinavam. Eu tinha bastante cuidado lá.* (Petúnia). *Fui muito bem atendida, muito bem preparada, tive suporte de psiquiatra, psicólogo, [...] quando eu vim fazer o pré-natal eles já me encaminharam, e daí foi supertranquilo. Tive o suporte dos dois, o psiquiatra e o psicólogo.* (Orquídea).

Diante de um momento delicado como a pandemia da COVID-19, os serviços de saúde precisaram se adaptar para atender às necessidades que emergiram; dentre elas está a falta de consultas médicas.

*Só tive a minha primeira consulta com eles, só passava pela triagem, não tinha médico pra atender por conta da COVID [...] eles marcavam para ter o acompanhamento, só que, daí, quando chegava lá no posto, os médicos estavam no UPA atendendo. Por conta da COVID. Porque era muita demanda e, daí, eles tinham que sair do posto para ir atender.* (Gérbera). *Durante a pandemia, não tinham tantas consultas disponíveis, tinha muita gente precisando, então era difícil conseguir. E para vir sempre tinha que ter cuidado, porque tinha gente pra fazer pré-natal, mas também tinha outros atendimentos, então a gente não sabia do risco que a gente tava correndo.* (Azaleia).

Além da redução da oferta de consultas médicas, a oferta de exames também foi afetada. A dificuldade de acesso a exames durante a gestação é um problema para acompanhar o desenvolvimento gestacional e coloca em risco o binômio. Ademais, alguns exames devem ser realizados respeitando a idade gestacional, e a ausência desses exames afeta a procura pelo cuidado, modificando os trajetos percorridos por gestantes na busca por cuidados em saúde.

*Eu abri o pré-natal e comecei a fazer os exames também, mas os exames eu não consegui pelo posto, aí acabei fazendo no particular.* (Azaleia). *Eu fiz um ultrassom particular. Você esperar pelo SUS demorava mais. E o acompanhamento pré-natal eu fiz no postinho mesmo.* (Lavanda).

De fato, as modificações impostas pela pandemia da COVID-19 alteraram o fluxo de atendimentos no acompanhamento pré-natal, desde os mais simples, como as vacinas, até os mais complexos, como o parto. A procura por cuidado se alterou, influenciada pela disponibilidade dos serviços de saúde em atender as gestantes, o que foi vivenciado de forma diferente por cada mulher.

*Eu consegui fazer as vacinas pelo SUS tranquilamente, mas não foi assim com aquela rapidez dos dias normais. [...] no SUS você tem que agendar, e tem uma demanda de gestantes. No dia do parto, no caso, eu ia ser atendida pelo plantonista. Então eu optei pelo plano de saúde, por eu conseguir ter essa facilidade da escolha do profissional que iria me acompanhar.* (Girassol). *Na minha primeira gestação eu fiz tudo no particular, mas, se eu fosse ter outro filho e eu tivesse a opção de escolher, eu escolhia o público. Eu fui muito melhor atendida aqui. Mesmo com a pandemia.* (Begônia).

Outro fator relevante foi a contaminação das gestantes e a busca dessas por cuidados médicos.

*No sétimo mês eu peguei COVID. A gente ficou bem assustado, com medo de acontecer alguma coisa com o bebê, de eu ter sintomas mais graves, de qual remédio que eu poderia tomar, por estar grávida, eu tinha a limitação de ficar tomando medicamento. Mas, daí, eu fiz a minha consulta obstétrica, o médico me recomendou alguns remédios mais sintomáticos mesmo.* (Gardênia).

A gestante, ao estar contaminada pelo SARS-CoV-2, pode apresentar desde sintomas gripais leves, como febre e tosse, até mais graves, como sintomas respiratórios, necessitando de cuidados médicos especializados. Além disso, a pandemia e o medo do contágio trouxeram repercussões psicológicas devido às restrições e incertezas impostas a essas mulheres. Assim, o itinerário terapêutico de mulheres que tiveram COVID-19 no período gestacional variou conforme a gravidade da doença e a disponibilidade dos serviços de saúde, visto que naquele momento muitas vezes havia um percurso pré-estabelecido.

*Eu estava com 37 semanas, eu fiquei internada na maternidade um pouco. Para verificar os batimentos cardíacos do bebê por conta da COVID-19. Estava tudo ok. Eles me mandaram pra casa. Eu fiquei tomando medicamento em casa.* (Petúnia). *Foi uma gravidez sadia, até 35 semanas [...] na quarta-feira eu fiz um teste. E deu positivo. Eu já senti um pouco de dificuldade de respirar. Mas eu achava comum. [...] eu tive a parada cardíaca, duas bactérias hospitalar, 45 dias entubada, 4 meses e meio de traqueostomia, 95% do pulmão afetado, e 53 dias no hospital.* (Magnólia).

É possível perceber que as dificuldades impostas pela pandemia da COVID-19 reduziram o acesso aos serviços de saúde, conferindo muitas fragilidades a um acompanhamento pré-natal de qualidade, uma vez que as mulheres realizaram menos consultas e exames. Consequentemente, tiveram oportunidades reduzidas para a educação em saúde e detecção precoce de possíveis problemas gestacionais.

### A espiritualidade como suporte terapêutico – subsistema cultural

Observou-se que as gestantes recorreram a suas crenças espirituais durante este período pandêmico, sendo este um suporte terapêutico para se sentirem fortalecidas quanto à contaminação e às adversidades impostas pela COVID-19.

*Eu sempre botei minha saúde em nome de Deus. Então, para mim, foi sempre firme na presença de Deus. No entanto, eu peguei a COVID, e fiquei para morrer. Mas Deus ali foi conforto, me curou.* (Petúnia). *Foi o que a gente fez aqui em casa, oramos, e estava propício a qualquer coisa, porque muita gente morreu, né, muita pessoa morreu, então a gente não sabia o que estava por vir.* (Hortênsia).

A espiritualidade das mulheres teve um papel importante em proporcionar ânimo e coragem para que elas procurassem os serviços de atendimento pré-natal em meio à pandemia. A religiosidade permitiu acreditar que a gestação aconteceria de forma saudável, mesmo durante o período pandêmico.

*A fé em Deus de ter a proteção e de necessitar, porque eu sabia que precisava, [...] eu precisava de uma ajuda profissional.* (Magnólia). *Foi uma coisa que eu achava que nunca mais ia engravidar, então, foi uma surpresa boa. Eu falei assim: não foi planejado por mim, mas foi planejado por Deus, porque ele sabia que eu precisava dele.* (Orquídea).

Percebe-se que a gestação estava relacionada a um planejamento maior, que a pandemia influenciou, e os desejos maternos não poderiam ser realizados.

*No meu dia que eu fui fazer o meu parto, eu estava na esperança que eu ia conseguir filmar o meu parto, era um sonho que eu tinha de poder filmar [...] eu liguei para o fotógrafo que eu tinha contratado, a gente tinha já deixado tudo certo, caso o hospital liberasse, ele iria entrar, [...] mas o hospital negou a entrada dele por conta do aumento de casos, [...] eu não consegui realizar esse desejo, que eu tinha muita vontade de filmar todo o meu parto, de ter as fotos de um profissional para eu poder eternizar. Então a pandemia, ela saiu do normal, e eu não consegui realizar alguns desejos que eu tinha, eu precisei me ausentar de muitos lugares que eu gostaria de ter ido durante a minha gestação.* (Margarida).

As experiências ao longo do caminho percorrido foram múltiplas, demonstrando-se boas e ruins, a depender da vivência de cada gestante.

## Discussão

Este estudo proporcionou a compreensão dos efeitos da pandemia de COVID-19 na dinâmica da busca do cuidado durante o período gestacional. Os achados indicam que a COVID-19 provocou alterações na dinâmica dos serviços de saúde, assim como na rede de apoio às gestantes, impactando alterações no IT vivenciado por elas.

Mesmo diante de tantas adversidades, foi possível observar a procura das mulheres pelo cuidado, tanto por meio dos serviços de saúde, como no âmbito familiar e cultural, o que demonstra certa capacidade adaptativa e a resiliência delas.

A procura das gestantes pelo apoio familiar, um elemento fundamental nas entrevistas, está vinculada à rede de apoio social, composta por pessoas significativas para elas, que oferecem suporte e reforço nas estratégias de enfrentamento diante das situações da vida. Essa rede inclui a família extensa, amigos, colegas de trabalho, relações comunitárias, serviços de saúde e até crenças religiosas ou políticas, englobando tanto as relações mais íntimas quanto as ocasionais^(17-18)^.

Seguindo essa perspectiva, Kleinman^(19)^ explica em sua teoria que os Sistemas de Cuidados à Saúde são fundamentados nas relações interpessoais, onde tudo está interligado. Pacientes, profissionais de saúde, doença e tratamento fazem parte desse contexto assistencial. As crenças e atitudes individuais são moldadas pelos papéis culturais. O autor destaca ainda que, para compreender as escolhas individuais, é essencial considerar as influências externas que afetam o Sistema de Cuidados à Saúde, como fatores ambientais, incluindo geografia, clima, demografia, questões sociais, poluição e o desenvolvimento agrícola e industrial^(19)^.

Os ITs dizem respeito aos cuidados à saúde que cada pessoa adota, sendo moldados pelas relações interpessoais. As escolhas de cuidados são influenciadas por três setores: a família, que exerce maior poder na definição do itinerário terapêutico; a cultura, que inclui profissionais não oficialmente reconhecidos, mas que compartilham as crenças sociais e valores culturais dos indivíduos; e os profissionais da saúde, que têm uma influência mais limitada, ao tenderem a priorizar o aspecto biológico no cuidado^(19)^.

Além disso, a teoria enfatiza a pluralidade do cuidado, argumentando que os indivíduos transitam entre os setores de cuidado (familiar, cultural e profissional) revelando a combinação de diferentes formas de cuidado, reforçando que o itinerário terapêutico não é linear e pode incluir tratamentos formais e informais de forma simultânea^(19)^.

O apoio social desempenha um papel crucial na prevenção da depressão pós-parto, evidenciando a importância de se dar atenção à qualidade da rede de apoio às gestantes durante o ciclo gravídico-puerperal. Nesse processo, não são somente a família, mas também os serviços de saúde, amigos e parceiros que assumem responsabilidades no suporte às mulheres nesse período^(20)^.

Nessa perspectiva, o objetivo da assistência pré-natal é garantir o desenvolvimento saudável da gestação, do parto e do puerpério, sem comprometer a saúde da mãe e do bebê. Isso envolve uma abordagem que considera também os aspectos psicossociais, além de atividades educativas e preventivas. Esse objetivo é uma responsabilidade compartilhada entre o médico e o enfermeiro^(8)^.

O profissional de saúde deve desenvolver um vínculo saudável com a gestante e sua família. Ao incentivar que essa rede de apoio compareça às consultas de acompanhamento pré-natal, tem-se em vista que é nesses momentos que as dúvidas são esclarecidas e há a possibilidade de todos se sentirem mais tranquilos e seguros com o processo gestacional^(21)^.

A enfermagem assume uma responsabilidade no cuidado à saúde dessas mulheres, ao desenvolver ações educativas pautadas na individualidade, integralidade, autocuidado e empoderamento feminino capacitando-as para tomar suas próprias decisões e contribuindo para sua independência quanto às demandas de saúde, estimulando-a a tornar-se corresponsável pelo próprio cuidado^(22)^.

Contudo, devido à pandemia de coronavírus, foi necessário realizar adaptações na atenção às gestantes, visando preservar a saúde tanto dessa população quanto dos profissionais de saúde envolvidos. Por apresentarem alterações fisiológicas no sistema imunológico, cardiorrespiratório e na coagulação, as gestantes em qualquer fase da gestação e as puérperas passaram a ser classificadas como parte do grupo de risco para a COVID-19, com maior chance de agravamento do quadro infeccioso, demandando, portanto, cuidados mais intensivos^(23)^.

Esses cuidados são necessários devido à alta transmissibilidade da doença, que ocorre por meio de gotículas respiratórias expelidas quando uma pessoa infectada tosse ou espirra, inaladas por indivíduos próximos. Também pode ocorrer através do contato pessoal, próximo ou com superfícies contaminadas, seguido do toque na boca, nariz e/ou olhos^(1)^.

Em resposta a essa situação, as autoridades de saúde implementaram medidas, visando reduzir a propagação do vírus, enfatizando a importância de manter a higiene das mãos, usar máscaras, reorganizar os atendimentos nos serviços de saúde e adotar o isolamento social^(24)^.

Por outro lado, o isolamento social, como forma prevenir a propagação da COVID-19, pôde fazer emergir sentimentos de solidão, uma vez que afastava a mulher de sua rede de apoio, que a auxiliava nas tarefas domésticas, orientação e suporte emocional^(25)^. Estudo realizado em 2020 revelou que as mulheres grávidas durante a pandemia de COVID-19 relataram níveis mais elevados de angústia e sintomas psiquiátricos em comparação às mulheres avaliadas antes da pandemia, com uma evolução para quadros de depressão e ansiedade. Os autores concluíram que a escassez de informações claras e confiáveis poderia agravar o risco de sofrimento psicológico e psicossocial entre essas gestantes^(26)^.

Implementar ferramentas de rastreio do risco de depressão na gravidez pode ser um passo importante para conhecer melhor as necessidades em saúde mental de mulheres inseridas na assistência pré-natal de risco habitual. Sabe-se que, muitas vezes, a saúde mental é negligenciada e, atrelado a isso, há que se considerar a dificuldade de acesso à assistência especializada nessa área na atenção primária à saúde^(27)^.

Em tempos de pandemia, a inovação e a criatividade passaram a fazer parte do cotidiano do profissional de saúde que, diariamente, enfrentava novos desafios. Foi preciso lançar mão de estratégias de adaptação para cumprir os calendários de saúde já existentes e que, nesse momento, precisavam ser executados de algum modo^(28)^.

Quanto à contaminação materna pelo coronavírus, existem registros de que as gestantes estão mais propensas a apresentar pré-eclâmpsia, diabetes gestacional e hipertensão, e síndrome HELLP^(29)^. Outro estudo demonstrou uma associação entre a infecção materna e desfechos fetais, tais como: parto prematuro, sofrimento fetal, baixo peso ao nascer, asfixia, ruptura prematura de membranas, coagulação intravascular disseminada e até morte fetal^(30)^.

As complicações decorrentes da gestação podem levar à hospitalização, o que, embora promova sensação de segurança e proteção, também desperta, para a gestante, sentimentos de angústia, dor, raiva, tristeza e medo oriundos da quebra de rotina e das intervenções e procedimentos realizados^(31)^.

O subsistema profissional, formado pelos serviços e profissionais de saúde, é organizado burocraticamente e com aprendizado e treinamento formal, sendo legalmente reconhecido, atraindo a sociedade por sua influência e capacidade de cura^(11)^.

Em relação aos aspectos culturais, a religião possui forte influência sobre a qualidade de vida em diferentes fases da vida, tornando-se relevante para promover saúde e prevenir agravos na população^(32)^. A dimensão espiritual no cuidado à saúde durante a gestação é um recurso fundamental para promover a autonomia, segurança e conforto, além de favorecer o fortalecimento do vínculo materno-fetal. A espiritualidade, assim, é um fenômeno que atribui significado a cada fase da vida, enquanto também confere propósito à própria existência humana^(33)^.

Ao buscar tanto o cuidado formal quanto o informal, diminui-se a distância entre esses sistemas, possibilitando práticas de cuidado que sejam multiculturais e significativas. Isso permite uma abordagem integral da saúde, que considera o cuidado cultural, ressaltando que, dessa forma, novos conhecimentos podem emergir, contribuindo para a transformação das práticas em saúde^(34)^.

As limitações do estudo se referem ao viés de memória das participantes entrevistadas devido ao tempo transcorrido entre a gestação e a coleta de dados para a pesquisa. Entretanto, ressalta-se que a gravidez se trata de um período muito marcante para a mulher e que, atrelada à pandemia, um evento histórico, possivelmente pouca ou nenhuma situação deve ter sido esquecida.

Os achados deste estudo são de extrema relevância, dadas as inúmeras alterações impostas aos serviços de saúde no período pandêmico, sendo algumas mantidas após a pandemia, como foi o caso das teleconsultas.

Além disso, os resultados poderão contribuir para a prática profissional daqueles que assistem à gestante no pré-natal, melhorando a sua compreensão acerca dos caminhos percorridos na busca pelo cuidado, bem como da necessidade de inclusão e aceitação dos familiares e aspectos culturais durante todo o acompanhamento pré-natal.

## Conclusão

O itinerário terapêutico percorrido por gestantes em busca do cuidado em saúde durante a pandemia da COVID-19 foi configurado em três subsistemas: familiar, profissional e cultural. As mulheres procuraram suporte primeiramente em suas famílias, na busca por afeto, carinho, orientação e apoio, e na sequência foram ao encontro do suporte profissional, sendo esse encontrado na Atenção Primária à Saúde, principal porta de entrada para o cuidado, durante todo o período gestacional, parto e puerpério. Muitas mulheres também abordaram, no contexto cultural, as crenças como instrumento de fortalecimento para o cuidado, ocorrendo de forma associada aos demais subsistemas.

Com a vivência da gestação durante a pandemia da COVID-19, os caminhos percorridos para a busca de cuidado foram ainda mais difíceis para essas gestantes, estando permeados por dúvidas e incertezas, além de uma nova configuração de rotinas e atendimentos adotados pelos serviços de saúde.

Muitas gestantes enfrentaram dilemas sobre procurar serviços de saúde durante a pandemia, logo, urge garantir o acesso contínuo aos cuidados pré-natais. Acredita-se que o presente estudo possa provocar a reflexão acerca da importância do cuidado individualizado e holístico para a gestante, buscando compreender e respeitar suas crenças e forma de viver, seu entendimento, angústias e necessidades momentâneas, a fim de qualificar a assistência pré-natal, especialmente em situações de adversidades, como foi a pandemia da COVID-19.

# Translation: Itinerario terapéutico de mujeres embarazadas durante la pandemia de COVID-19: una mirada desde los sistemas de atención sanitaria*

**DOI:** 10.1590/1518-8345.7623.4628

**Authors:** Alana Vitória Escritori Cargnin, Camila Moraes Garollo Piran, Beatriz Sousa da Fonseca, Camila Siqueira Floresta Lehmkuhl, Maria de Fátima Garcia Lopes Merino, Adriana Zilly, Marcela Demitto Furtado

**Affiliations:** 1 Universidade Estadual de Maringá, Maringá, PR, Brasil.; 2 Becaria de la Coordenação de Aperfeiçoamento de Pessoal de Nível Superior (CAPES), Brasil.; 3 Universidade Estadual de Maringá, Departamento de Enfermagem, Maringá, PR, Brasil.; 4 Universidade do Oeste do Paraná, Departamento de Enfermagem, Foz do Iguaçu, PR, Brasil.

**Keywords:** COVID-19, Atención Prenatal, Embarazo, Ruta Terapéutica, Modelos de Atención de Salud, Salud de la Mujer

## Abstract

comprender el itinerario terapéutico de las mujeres que transitaron el embarazo durante la pandemia de COVID-19.

estudio descriptivo y exploratorio con enfoque cualitativo, desarrollado con 16 mujeres que transitaron su embarazo durante la COVID-19. Se utilizó como marco teórico el Modelo de Sistemas de Atención de Salud, propuesto por Arthur Kleinman. Se realizaron entrevistas semiestructuradas, grabadas en audio, transcriptas en su totalidad y sometidas al análisis de contenido propuesto por Bardin y al *software* IRaMuTeQ.

participaron en el estudio mujeres cuyas edades oscilaron entre 20 y 47 años. La mayoría de los participantes eran blancas y casadas. Del análisis de contenido surgieron tres categorías: 1- Principales redes de apoyo durante el embarazo ― subsistema familiar; 2- Seguimiento durante el embarazo ― subsistema profesional; y 3- La espiritualidad como soporte terapéutico ― subsistema cultural.

se observó que los caminos recorridos por las embarazadas en búsqueda de atención sanitaria durante la pandemia de COVID-19 se volvieron aún más difíciles, atravesados por dudas e incertidumbres generadas por la enfermedad, así como por las nuevas configuraciones de rutinas y cuidados que adoptaron los servicios de salud.

## Destacados

**(1)** El itinerario terapéutico se vio influenciado por los cambios impuestos a los servicios de salud. **(2)** El miedo al contagio dificultó la búsqueda de atención sanitaria. **(3)** El sistema sanitario permitió conocer las principales redes de apoyo. **(4)** La espiritualidad sirvió de soporte ante las dificultades que impuso la pandemia.

## Introducción

El 11 de marzo de 2020, la Organización Mundial de la Salud declaró una nueva pandemia, causada por el nuevo coronavirus, llamada *Severe Acute Respiratory Syndrome Coronavirus* 2 (SARS-CoV-2), responsable de causar la enfermedad COVID-19^(1)^. Debido a la gravedad y rápida propagación de la enfermedad se definieron grupos de riesgo, uno de ellos constituido por mujeres embarazadas^(2)^. Aunque la mayoría de las embarazadas diagnosticadas con COVID-19 presentaron síntomas clínicos leves a moderados, se advierte un mayor riesgo de complicaciones maternas en presencia de comorbilidades asociadas, como trastornos hipertensivos y obesidad^(3)^.

El riesgo de mortalidad materna es 22 veces mayor en mujeres embarazadas infectadas con COVID-19, en regiones con menor acceso a servicios de salud y cuando no se hallan disponibles los servicios integrales de las Unidades de Cuidados Intensivos (UCI)^(4)^.

La mortalidad materna en embarazadas con COVID-19 puede estar relacionada con factores socioeconómicos, dificultades de acceso a los servicios de salud y fallas en la organización del sistema, que se agravan por la saturación hospitalaria. Por otra parte, los ámbitos con atención obstétrica bien estructurada y disponibilidad adecuada de recursos para UCI materna presentan tasas de mortalidad más bajas^(4)^.

El embarazo provoca varios cambios fisiológicos en el cuerpo de la mujer, incluidos cambios en la postura, el metabolismo y los sistemas cardiovascular, sanguíneo y urinario. También se producen cambios en el gasto cardíaco, en la concentración y elevación de ciertas sustancias, además de adaptaciones en los sistemas respiratorio, digestivo y endocrino. En los genitales se observan transformaciones en la vulva, vagina y útero. Además, existen implicaciones clínicas asociadas a cambios cardiovasculares, sanguíneos, urinarios, respiratorios, digestivos y endocrinos^(5)^.

Con la llegada del COVID-19, los servicios de salud se vieron en la necesidad de reorganizar el proceso de trabajo, articular estrategias de control y prevención de enfermedades y fortalecer toda la red sanitaria, con el fin de brindar a los usuarios la seguridad y el soporte necesario^(6)^.

El Ministerio de Salud de Brasil (MS) recomienda realizar al menos seis consultas durante la atención prenatal. Sin embargo, a raíz de las nuevas medidas sanitarias para controlar el virus, destinadas a proteger a las mujeres embarazadas de la exposición y el riesgo de contagio en las unidades sanitarias, las consultas presenciales se volvieron más espaciadas que lo recomendado anteriormente^(7)^.

Con el objetivo de reducir la circulación de usuarios en los ambientes y dar continuidad a la atención de rutina en las unidades de salud, los servicios de teleconsulta asociados a las consultas presenciales surgen como estrategia para el cuidado de gestantes durante la atención prenatal. También se realizó el seguimiento de mujeres embarazadas que presentaron síntomas de COVID-19. Esta medida fue evaluada constantemente por el equipo, de conformidad con los protocolos de recomendación de las entidades de salud^(8)^.

Incluso en situaciones complejas, como una pandemia, resulta importante brindar acceso a información de calidad durante el período gestacional, para vivir con plenitud este proceso y tener autonomía en la toma de decisiones que involucran el ciclo embarazo-puerperio. Además, en este contexto, es fundamental ofrecer una asistencia individualizada que valore también los aspectos psicosociales. Y, para comprender mejor la experiencia de estas futuras madres, es necesario conocer los caminos que han recorrido en la búsqueda de atención para resolver sus problemas de salud durante la pandemia de COVID-19^(9)^, proceso este que se define como Itinerario Terapéutico (IT).

El concepto de IT cobró importancia cuando el psiquiatra y antropólogo Arthur Kleinman enfatizó la influencia cultural en las elecciones del paciente respecto a la vía de atención, lo que se denominó modelo teórico de Sistemas de Atención de Salud. Este modelo se refiere a los ámbitos donde los individuos buscan soluciones a sus problemas de salud, clasificados en tres subsistemas: familiar, profesional y cultural^(10-11)^.

En este contexto, los Itinerarios Terapéuticos (IT) representan una estrategia de investigación que permite comprender los caminos, dinámicas y relaciones que establecen las personas en su búsqueda de atención de salud. Los IT se configuran como un conjunto de interacciones y prácticas construidas por grupos sociales para ampliar las posibilidades de acceso y asistencia sanitaria^(11)^.

La velocidad con la que se propagó la pandemia de COVID-19 en el mundo ha sido devastadora y ha afectado a distintos públicos y grupos de edad, volviéndose especialmente grave para la población más vulnerable, como las mujeres embarazadas. Las dificultades para acceder a los servicios y el aislamiento de los centros de apoyo provocan en las mujeres embarazadas problemas de salud física y mental que pueden afectarlas a ellas y al feto. Esas dificultades evidencian necesidades y debilidades, además de exacerbar las desigualdades sociales, sanitarias y económicas, por lo que es necesario comprender las demandas en los ámbitos personal, cultural y sanitario, a fin de promover y realizar eficazmente la atención integral a la salud. A tal efecto, se plantea el siguiente interrogante: ¿Qué itinerario terapéutico siguieron las mujeres embarazadas durante la pandemia de COVID-19?

En este contexto, el objetivo fue comprender el itinerario terapéutico de las mujeres que transitaron el embarazo durante la pandemia de COVID-19.

## Método

### Diseño del estudio

Estudio descriptivo y exploratorio con enfoque cualitativo, a partir del marco teórico del Modelo de Sistemas de Atención de Salud propuesto por Arthur Kleinman^(10)^.

Este marco teórico aborda los espacios donde los individuos buscan soluciones a sus problemas de salud, y está compuesto por tres subsistemas: el familiar, el profesional y el cultural (el profesional constituido por las prácticas médicas formales, como la biomedicina y la homeopatía; el popular, que es el cuidado domiciliario y el autocuidado; o el *folk*, que corresponde a las prácticas de cuidado místico y religioso)^(10-11)^.

Con el fin de garantizar la calidad y transparencia de la redacción, se aplicaron los criterios previstos por el *Consolidated Criteria for Reporting Qualitative Research (COREQ)*.

### Escenario y período de recopilación de datos

Se realizó un sorteo aleatorio, en el que se seleccionaron diez Unidades Básicas de Salud (UBS) para la recolección de datos. La recolección de datos se llevó a cabo entre junio y octubre de 2023, mediante entrevistas semiestructuradas.

El contacto con los participantes se produjo a través del suministro de datos por parte de los servicios de salud y mediante el contacto directo con las mujeres que tenían turnos programados durante el período seleccionado para la investigación. Después de la aceptación, el día y la hora fueron programados según la disponibilidad del participante, y las entrevistas fueron realizadas por la enfermera investigadora, en el domicilio del participante o en espacios facilitados por la UBS según la preferencia del participante, garantizando siempre su completa privacidad.

### Participantes y criterios de selección

Participaron en el estudio mujeres que transitaron su embarazo durante la pandemia de COVID-19, residentes en el municipio de Maringá, ciudad ubicada en el interior de Paraná, Brasil, y que se atendían en una de las 34 UBS del municipio.

El proceso de identificación de estas mujeres se realizó a través del sistema UBS, mediante una búsqueda que incluía a todos los niños nacidos durante el período de la pandemia. De estos, se consideraron aquellos en los que el período gestacional materno ocurrió entre marzo de 2020 y junio de 2021, considerando la pandemia en Brasil y la flexibilización de las medidas restrictivas contra el COVID-19 en el referido país^(12)^.

Los criterios de inclusión fueron: mujeres que estuvieron embarazadas entre marzo de 2020 y junio de 2021, registradas en la UBS seleccionada en el momento de la entrevista y que iniciaron atención prenatal en la red pública, más allá de su lugar de residencia en el momento de la gestación. Se excluyeron las mujeres menores de 18 años y aquellas con algún déficit cognitivo que imposibilitara la comprensión de las preguntas de la entrevista.

### Instrumento y procedimientos para la recolección de datos

Antes de iniciar cada entrevista se procedió a la lectura del Formulario de Consentimiento Libre e Informado (FCLI) y se solicitó permiso para la grabación, además de aportar datos sobre la investigación y aclarar eventuales dudas.

Las entrevistas fueron realizadas por una enfermera investigadora con experiencia previa en investigación científica, a partir de la siguiente pregunta guía: “Cuéntame cómo fue para ti estar embarazada durante la pandemia de COVID-19”. Además, hubo otras consignas orientadoras, previamente delimitadas por la investigadora, las cuales estaban relacionadas con el descubrimiento del embarazo, las medidas restrictivas impuestas a las embarazadas, las dificultades/facilidades encontradas para acceder a los servicios de salud y en relación a los servicios de salud frecuentados durante la pandemia, entre otros aspectos relacionados con el embarazo y el período pandémico. Estos puntos se incluyeron con el fin de lograr el objetivo del estudio. También se aplicó un cuestionario sociodemográfico, con ítems como: edad, raza/color, número de hijos e ingresos para conocer las características de los participantes.

No hubo rechazos a participar en el estudio y el número final de participantes se estableció durante las entrevistas en las que se alcanzó la saturación teórica: dada la similitud de significados durante las entrevistas y no habiendo más información nueva, se interrumpió la recolección de datos y se estableció el número de participantes^(13)^.

### Procesamiento y análisis de datos

Todas las entrevistas fueron grabadas y luego transcriptas en su totalidad, con una duración promedio de 22 minutos. El material fue codificado con el auxilio del *software.* IRaMuTeQ y el tratamiento de los resultados se llevó a cabo por el análisis temático propuesto por Bardin^(14)^. En este proceso participaron tres investigadores que realizaron el análisis de forma independiente. No hubo conflictos en la interpretación de los resultados. El corpus para el análisis se obtuvo luego de la transcripción literal, adaptación lingüística y estructuración estandarizada de los textos, asegurando así la confiabilidad de los datos.

El análisis de contenido se dividió en tres etapas: 1) reconocimiento de todo el material seguido de la sistematización y codificación de los datos; 2) la incorporación de datos brutos en unidades homogéneas que faciliten la descripción y caracterización del contenido, para luego organizarlos en unidades de significado; 3) inferencia sobre datos previamente presentes en la literatura sobre el tema, asociados a los resultados encontrados^(15)^.

A continuación, se realizó la triangulación de datos, combinando diferentes estrategias de análisis para reforzar la validez de los hallazgos. Entre ellos, se utilizó el análisis de similitud, basado en la teoría de grafos, que permite identificar coocurrencias entre palabras y sus conexiones en el corpus textual. Este tipo de análisis contribuye a la comprensión de la estructura del contenido y permite visualizar patrones comunes y especificidades del material, lo que fortalece la interpretación de los datos^(15-16)^.

### Aspectos éticos

El proyecto de investigación fue aprobado por el Comité de Ética en Investigación bajo Dictamen n.°4.886.085 (Certificado de Presentación de Apreciación Ética - CAAE: 39060120.1.3004.0104) y todos los participantes firmaron el Formulario de Consentimiento Libre e Informado (FCLI), de acuerdo con la Resolución n.º 466/12 del Consejo Nacional de Salud. Para garantizar el anonimato de los participantes, los nombres de los entrevistados fueron sustituidos por nombres de flores.

## Resultados

Dieciséis mujeres de entre 20 y 47 años participaron en el estudio. La mayoría de las participantes eran blancas (56,3%), casadas (68,8%) y multíparas (62,5%), con ingresos que oscilaban entre uno y cinco salarios mínimos, y, en gran parte, ganaban entre uno y dos salarios (56,3%).

A través de la triangulación de datos, se observó la similitud de palabras en su estructura, núcleo central y sistema periférico de los contenidos de los menús analizados, habiéndose identificado tres grandes ejes organizadores centrados en los recorridos transitados por las gestantes en la búsqueda de atención durante el período de pandemia, como se muestra en la Figura 1.

**Figura 1 -:** Similitud de palabras extraídas de los menús del itinerario terapéutico para embarazadas durante la pandemia de COVID-19. Maringá, PR, Brasil, 2023

A partir de la lectura exhaustiva y triangulación de los datos, se percibió que la experiencia de las mujeres que vivieron el embarazo durante la pandemia estuvo marcada por un arduo recorrido en la búsqueda de cuidados, lo que permitió construir tres categorías, descritas a continuación.

### Principales redes de apoyo durante el embarazo: subsistema familiar

La palabra “estar” está muy relacionada con el embarazo, “estar embarazada”, y se descubrió que, al darse cuenta de que estaban embarazadas, las mujeres buscaban a sus familiares o personas cercanas. Así, la primera búsqueda de cuidados se produjo al compartir la noticia del embarazo con alguien, con la perspectiva, aunque inconsciente, de encontrar afecto, apoyo, consuelo y compañía para transitar todo el recorrido del embarazo en un momento de tanta incertidumbre, como la pandemia de COVID-19.

*Mi marido estaba conmigo cuando hice la prueba, así que nos enteramos juntos.* (Tulipán). *Primero hablé con mi madre y luego ella me aconsejó ir al centro de salud.* (Azalea). *La primera persona fue mi sobrina, la llamé llorando [...].* (Violeta).

La presencia familiar durante el embarazo se vio dificultada por las restricciones impuestas para reducir el contagio, ya sea durante las consultas o por la sensación de menor apoyo recibido a lo largo del embarazo. La experiencia de las mujeres embarazadas que buscaban asistencia en los servicios de salud junto a sus familias se vio alterada debido a las adversidades generadas por la pandemia de COVID-19.

*Mi madre sólo llegó cuando iba a tener el bebé. Sin la pandemia mis hermanas y mi madre hubieran podido venir, pero luego todo cerró, no pude viajar. Entonces creo que sin la pandemia hubiera tenido más apoyo.* (Lirio). *Mi familia quería acompañarme y no pudo, así que tuve que elegir entre mi marido u otro familiar.* (Gardenia). *Mi familia, como bloque, es muy participativa en algunos aspectos. Muy acogedora. Pero debido a la pandemia, nos alejamos.* (Margarita).

Además del apoyo emocional brindado por la familia, durante la pandemia las mujeres embarazadas necesitaron apoyo económico debido a las restricciones impuestas, que cambiaron su rutina laboral, sumado al hecho de que muchas embarazadas se habían apartado del ejercicio profesional por el riesgo de contraer COVID-19.

*Mis tías me ayudaron en todo, en cuanto a la alimentación, tuve mucho apoyo, incluso algo de apoyo económico.* (Rosa). *Pasamos por un período terrible en términos económicos, no teníamos soporte financiero. Yo no tenía sueldo y él estaba desempleado [...] Pedí la canasta básica de alimentos porque tenía que criar a los niños pequeños.* (Hortensia). *Las mujeres embarazadas no podían trabajar y eso me afectó mucho.* (Margarita).

Las mujeres también buscaban amigos o personas con las que se sentían cercanas, ya sea porque su familia estaba ausente o porque se sentían más cómodas en presencia de sus amigos.

*Entonces, primero se lo conté a una amiga mía, hablamos mucho y lloramos juntas.No tengo muchos familiares aquí, pero he tenido total apoyo de mis amigos.* (Orquídea). *Mis profesores también me ayudaron mucho. Tuve una profesora a la que le hice muchas preguntas y me ayudó mucho.* (Gardenia).

La participación de la familia, así como la de un amigo cercano, durante el embarazo, hace que la experiencia sea más liviana y tranquila y el apoyo puede darse como soporte emocional o financiero. Se advierte que las mujeres quieren sentirse más seguras y cómodas en presencia de alguien conocido durante el embarazo.

### Atención prenatal – subsistema profesional

Las palabras “ir” y “hacer” transmiten la búsqueda de servicios de salud, esenciales para un embarazo saludable. Por ello, se recomienda iniciar lo antes posible la atención prenatal, para el seguimiento y la valoración materno-fetal, que deben ser completos, con al menos seis consultas, para cubrir todas las necesidades que puedan surgir durante la gestación.

*Fui con mi actual esposo, luego llegué al centro de salud y comencé el control prenatal. Yo tomaba medicación para la depresión y me derivaron a alto riesgo.* (Rosa). *Me tomó unos días llegar aquí y comenzar este seguimiento prenatal. También porque tuve un aborto en mi primer embarazo, entonces preferí hacerme primero una ecografía, para estar segura de que tenía un bebé, y luego venir aquí para empezar con el control prenatal. Y luego hice todo el seguimiento prenatal en el centro de salud.* (Anturio).

Una buena atención prenatal crea un vínculo entre el equipo de salud y la mujer embarazada, posibilita una buena relación y facilita el proceso de atención. Esta proximidad será favorable no sólo al embarazo, sino también al período posparto, en el cual las necesidades sanitarias permanecen evidentes.

*Yo recibí atención especial porque tuve un embarazo de alto riesgo. Entonces iba a dos unidades de salud. Al centro de salud y a otra unidad cuyo nombre no recuerdo. Pero allí me trataron muy bien y me examinaron. Allí recibí muchos cuidados.* (Petunia). *Fui muy bien atendida, muy bien preparada, tuve el soporte de un psiquiatra, de un psicólogo, [...] cuando vine a control prenatal ya me derivaron, y de ahí en más fue súper fácil. Tuve el apoyo de ambos, el psiquiatra y el psicólogo.* (Orquídea).

Ante un momento delicado como la pandemia de COVID-19, los servicios de salud debieron adaptarse para satisfacer las necesidades que se presentaban. Entre ellas, está la falta de citas médicas.

*Solo tuve mi primera consulta con ellos, solo pasé por el tamizaje, no había médico que me atendiera por el COVID [...] me citaron para seguimiento, pero luego, cuando llegué al centro médico, los médicos estaban en la UPA atendiendo. Eso por el COVID. Porque había mucha demanda y por tanto tenían que salir del centro para atender la demanda.* (Gerbera). *Durante la pandemia no había tantos turnos disponibles, muchas lo necesitaban, por eso era difícil conseguirlos. Y para venir, siempre debíamos tener cuidado, porque había gente para el prenatal, pero también había otros servicios, entonces no sabíamos el riesgo que corríamos.* (Azalea).

Además de la reducción en la oferta de consultas médicas, también se vio afectada la oferta de exámenes. La dificultad para acceder a las pruebas durante el embarazo supone un problema para el seguimiento del desarrollo gestacional y pone en riesgo al binomio. Además, se deben realizar algunas pruebas respetando la edad gestacional, y la ausencia de estas pruebas afecta la búsqueda de atención, modificando los caminos que toman las embarazadas en la búsqueda de atención médica.

*Comencé con el control prenatal y empecé a hacerme los exámenes también, pero no pude hacérmelos a través del centro de salud, así que terminé haciéndolos de manera privada.* (Azalea). *Me hice una ecografía privada. Si esperabas para hacerla por el SUS tardaba* más. *Pero el seguimiento prenatal sí lo hice en el centro de salud.* (Lavanda).

De hecho, los cambios impuestos por la pandemia de COVID-19 han alterado los flujos de atención prenatal, desde los más simples, como las vacunaciones, hasta los más complejos, como el parto. La demanda de atención sanitaria se modificó, influenciada por la disponibilidad de servicios sanitarios para atender a las mujeres embarazadas, y esta situación ha sido vivida de manera diferente por cada mujer.

*Pude conseguir las vacunas a través del SUS sin ningún problema, pero no fue tan rápido como suele ser. [...] en el SUS hay que programar una consulta y hay demanda por parte de embarazadas. El día del nacimiento, en este caso, me iba a atender el médico de turno. Entonces opté por el plan de salud, porque podía tener la facilidad de elegir al profesional que me acompañaría.* (Girasol). *Durante mi primer embarazo hice todo de forma privada, pero si tuviera otro hijo y tuviera la opción de elegir, elegiría la asistencia* pública. Aquí me trataron mucho mejor. Incluso con la pandemia. (Begonia).

Otro factor relevante fue el contagio de mujeres embarazadas y su búsqueda de atención médica.

*En el séptimo mes me contagié de COVID. Estábamos muy asustados. Tenía miedo de que le pasara algo al bebé, de que yo tuviera síntomas más graves y de no saber qué medicamento podía tomar, porque estaba embarazada y tenía una limitación para tomar medicamentos. Pero luego tuve mi cita con el obstetra y el médico me recomendó algunos medicamentos más sintomáticos.* (Gardenia).

Las mujeres embarazadas, al infectarse con SARS-CoV-2, pueden presentar síntomas leves parecidos a los de la gripe, como fiebre y tos, incluso más graves, como síntomas respiratorios, que requieren atención médica especializada. Además, la pandemia y el miedo al contagio han tenido repercusiones psicológicas debido a las restricciones e incertidumbres impuestas a estas mujeres. Así, el itinerario terapéutico de las mujeres que tuvieron COVID-19 durante el embarazo varió según la gravedad de la enfermedad y la disponibilidad de servicios de salud, ya que en ese momento muchas veces había un camino preestablecido.

*Tenía 37 semanas de embarazo y permanecí internada en la sala de maternidad por un tiempo. Para verificar los latidos del corazón del bebé debido al COVID-19. Todo estuvo bien. Me enviaron a casa. Me quedé en casa, tomando medicamentos.* (Petunia). *Fue un embarazo saludable, hasta las 35 semanas [...] el miércoles me hice un test. Y salió positivo. Ya he sentido una pequeña dificultad para respirar. Pero pensé que era común. [...] Tuve un paro cardíaco, dos infecciones hospitalarias, 45 días entubada, 4 meses y medio con traqueotomía, 95% de mis pulmones afectados, y 53 días en el hospital.* (Magnolia).

Es posible advertir que las dificultades impuestas por la pandemia de COVID-19 redujeron el acceso a los servicios de salud, y generaron el debilitamiento de la calidad de la atención prenatal, ya que las mujeres asistieron a menos consultas y exámenes. En consecuencia, tenían menos oportunidades de recibir educación sanitaria y de detectar tempranamente posibles problemas gestacionales.

### La espiritualidad como soporte terapéutico – subsistema cultural

Se observó que las mujeres embarazadas recurrieron a sus creencias espirituales durante este período de pandemia, lo que constituyó un soporte terapéutico para sentirse fortalecidas ante el contagio y las adversidades impuestas por el COVID-19.

*Siempre he puesto mi salud en manos de Dios. Para mí fue siempre permanecer firme en la presencia de Dios. Sin embargo, me contagié de COVID y estaba a punto de morir. Pero Dios estaba allí para consolarme, me sanó.* (Petunia). *Eso fue lo que hicimos aquí en casa, oramos, y fue propicio para cualquier cosa, porque murió mucha gente, ¿no cierto?, murió mucha gente, entonces no sabíamos lo que se venía.* (Hortensia).

La espiritualidad de las mujeres jugó un papel importante al brindarles aliento y coraje para buscar servicios de atención prenatal en medio de la pandemia. La religiosidad nos permitió creer que el embarazo se desarrollaría de forma saludable, incluso en tiempos de pandemia.

*La fe en Dios para tener protección y necesitarla, porque sabía que la necesitaba, [...] necesitaba ayuda profesional.* (Magnolia). *Fue algo que pensé que nunca volvería a quedar embarazada, así que fue una grata sorpresa. Dije: no fue planeado por mí, sino por Dios, porque Él sabía que yo lo necesitaba.* (Orquídea).

Se evidencia que el embarazo estaba relacionado con un plan mayor en el que la pandemia se interpuso y los deseos de la madre no se pudieron cumplir.

*El día que fui a dar a luz, tenía la esperanza de poder filmar mi parto, era un sueño que tenía de poder filmar [...] Llamé al fotógrafo que había contratado, ya habíamos arreglado todo, si el hospital lo permitía, entraría, [...] pero el hospital le negó la entrada por el aumento de casos, [...] No pude cumplir ese deseo, que tenía muchas ganas de filmar todo mi parto, que me tomaran las fotos un profesional para poder inmortalizarlas. Luego llegó la pandemia, fue algo fuera de lo común y no pude cumplir algunos de mis deseos, tuve que alejarme de muchos lugares a los que me hubiera gustado ir durante mi embarazo.* (Margarita).

Las experiencias a lo largo del camino fueron múltiples, tanto buenas como malas, y variaban según la experiencia de cada embarazada.

## Discusión

Este estudio proporcionó una comprensión de los efectos de la pandemia de COVID-19 en la dinámica de la búsqueda de atención durante el período gestacional. Los hallazgos indican que el COVID-19 provocó cambios en la dinámica de los servicios de salud, así como en la red de apoyo a las mujeres embarazadas, lo que implicó cambios en los IT transitados por ellas.

Aún frente a tantas adversidades, fue posible observar que las mujeres buscaban atención, tanto a través de los servicios de salud como dentro del contexto familiar y cultural, lo que demuestra cierta capacidad de adaptación y su resiliencia.

La búsqueda de apoyo familiar por parte de las mujeres embarazadas, elemento fundamental en las entrevistas, se vincula con la red de apoyo social, conformada por personas significativas para ellas, que brindan apoyo y refuerzo en las estrategias de afrontamiento ante situaciones de la vida. Esta red incluye familia extendida, amigos, compañeros de trabajo, relaciones comunitarias, servicios de salud e incluso creencias religiosas o políticas, y abarca tanto a las relaciones más íntimas como a las ocasionales^(17-18)^.

Siguiendo esta perspectiva, Kleinman^(19)^ explica en su teoría que los Sistemas de Salud se basan en relaciones interpersonales en las que todo está interconectado. Los pacientes, los profesionales sanitarios, la enfermedad y el tratamiento forman parte de este contexto asistencial. Las creencias y actitudes individuales están determinadas por los roles culturales. El autor destaca además que, para comprender las elecciones individuales, es esencial considerar las influencias externas que afectan al Sistema de Atención de Salud, como los factores ambientales, incluidos el clima, la geografía, la demografía, las cuestiones sociales, la contaminación y el desarrollo agrícola e industrial^(19)^.

Los IT se refieren a la atención sanitaria que cada persona adopta y que está condicionada por las relaciones interpersonales. Las decisiones de cuidado están influenciadas por tres sectores: la familia, que ejerce mayor poder en la definición del itinerario terapéutico; la cultura, que incluye a los profesionales que no están oficialmente reconocidos pero que comparten las creencias sociales y los valores culturales de los individuos; y los profesionales de la salud, quienes ejercen una influencia más acotada, ya que tienden a priorizar el aspecto biológico en la atención^(19)^.

Además, la teoría enfatiza la pluralidad del cuidado, bajo el argumento de que los individuos se mueven entre sectores de cuidado (familiar, cultural y profesional) y revela la combinación de diferentes formas de cuidado, además de reforzar que el itinerario terapéutico no es lineal y puede incluir, al mismo tiempo, tratamientos formales e informales^(19)^.

El apoyo social juega un papel crucial en la prevención de la depresión posparto, destacándose la importancia de prestar atención a la calidad de la red de apoyo a la mujer embarazada durante el ciclo embarazo-puerperio. En este proceso, no sólo la familia, sino también los servicios de salud, los amigos y la pareja asumen responsabilidades en el apoyo a las mujeres durante este período^(20)^.

Desde esta perspectiva, el objetivo de la atención prenatal es asegurar el desarrollo saludable del embarazo, parto y puerperio, sin comprometer la salud de la madre y del bebé. Se trata de un enfoque que también considera aspectos psicosociales, además de actividades educativas y preventivas. Este objetivo es una responsabilidad compartida entre el médico y el enfermero^(8)^.

El profesional de la salud debe desarrollar un vínculo saludable con la mujer embarazada y su familia. Al incentivar la presencia de esta red de apoyo en las consultas de atención prenatal, se entiende que en estos momentos se aclaran dudas y se abre la posibilidad de que todos se sientan más tranquilos y seguros sobre el proceso gestacional^(21)^.

La enfermería asume la responsabilidad por el cuidado de la salud de estas mujeres, al desarrollar acciones educativas basadas en la individualidad, la integralidad, el autocuidado y el empoderamiento femenino, lo que les permite tomar sus propias decisiones y contribuye a su independencia frente a las demandas sanitarias, además de estimularlas a ser corresponsables de su propio cuidado^(22)^.

Sin embargo, debido a la pandemia de coronavirus, fue necesario realizar ajustes en la atención brindada a las mujeres embarazadas, con el objetivo de preservar la salud tanto de esta población como de los profesionales de la salud implicados. Debido a los cambios fisiológicos en los sistemas inmune, cardiorrespiratorio y de coagulación, las mujeres embarazadas en cualquier etapa de la gestación y las puérperas comenzaron a formar parte del grupo de riesgo para COVID-19, con mayor probabilidad de agravamiento del cuadro infeccioso, por lo que requerían cuidados más intensivos^(23)^.

Estas precauciones son necesarias debido a la alta transmisibilidad de la enfermedad, producida a través de las gotitas respiratorias expulsadas cuando una persona infectada tose o estornuda, que son inhaladas por individuos cercanos. También puede ocurrir a través del contacto personal, cercano, o con superficies contaminadas y luego tocarse la boca, la nariz y/o los ojos^(1)^.

Ante esta situación, las autoridades sanitarias implementaron medidas encaminadas a reducir la propagación del virus, enfatizando la importancia de mantener la higiene de manos, el uso de mascarillas, reorganizar la atención de los servicios de salud y adoptar el aislamiento social^(24)^.

Por otra parte, el aislamiento social, como forma de prevenir la propagación del COVID-19, puede haber generado sentimientos de soledad, ya que apartó a las mujeres de su red de contención, que las auxiliaba con las tareas del hogar, la orientación y el soporte emocional^(25)^. Un estudio realizado en 2020 reveló que las mujeres embarazadas durante la pandemia de COVID-19 reportaron mayores niveles de angustia y síntomas psiquiátricos en comparación con las mujeres evaluadas antes de la pandemia, con evolución hacia la depresión y la ansiedad. Los autores concluyeron que la falta de información clara y confiable podría agravar el riesgo de angustia psicológica y psicosocial entre estas mujeres embarazadas^(26)^.

La implementación de herramientas para detectar el riesgo de depresión durante el embarazo puede ser un paso importante para conocer las necesidades de salud mental de las mujeres que reciben atención prenatal de bajo riesgo. Se sabe que, con frecuencia, se descuida a la salud mental, y, a este respecto, es necesario considerar las dificultades para acceder a asistencia especializada en esta área en la atención primaria de salud^(27)^.

En tiempos de pandemia, la innovación y la creatividad pasaron a formar parte del día a día de los profesionales de la salud que a diario afrontaban nuevos retos. Fue necesario utilizar estrategias de adaptación para cumplir con los calendarios de salud existentes que, en ese momento, debían cumplimentarse de alguna manera^(28)^.

Respecto a la contaminación materna por coronavirus, existen registros de que las embarazadas tienen mayor probabilidad de presentar preeclampsia, diabetes gestacional, hipertensión y síndrome HELLP^(29)^. Otro estudio demostró una asociación entre la infección materna y resultados fetales, como: nacimiento prematuro, sufrimiento fetal, bajo peso al nacer, asfixia, ruptura prematura de membranas, coagulación intravascular diseminada e incluso muerte fetal^(30)^.

Las complicaciones derivadas del embarazo pueden llevar a la hospitalización, que si bien promueve una sensación de seguridad y protección, también despierta en la embarazada sentimientos de angustia, dolor, rabia, tristeza y miedo derivados de la alteración de la rutina y de las intervenciones y procedimientos realizados^(31)^.

El subsistema profesional, formado por los servicios y profesionales de salud, está organizado de manera burocrática, con capacitación y aprendizaje formal. Es reconocido legalmente y atrae a la sociedad por su influencia y capacidad curativa^(11)^.

En relación con los aspectos culturales, la religión tiene una fuerte influencia en la calidad de vida en las diferentes etapas de la vida, volviéndose relevante para la promoción de la salud y la prevención de enfermedades en la población^(32)^. La dimensión espiritual en la atención de la salud durante el embarazo es un recurso fundamental para promover la autonomía, la seguridad y el confort, además de favorecer el fortalecimiento del vínculo materno-fetal. La espiritualidad, por ende, es un fenómeno que da sentido a cada etapa de la vida, a la vez que confiere un propósito a la propia existencia humana^(33)^.

Al buscar atención, ya sea formal o informal, se achica la distancia entre estos sistemas, lo que conlleva a prácticas de atención multiculturales y significativas. Esto permite un abordaje integral de la salud que considera el cuidado cultural. De este modo, pueden surgir nuevos conocimientos que contribuyan a la transformación de las prácticas de sanitarias^(34)^.

Las limitaciones del estudio se refieren al sesgo de memoria de las participantes entrevistadas debido al tiempo transcurrido entre el embarazo y la recolección de datos para la investigación. Sin embargo, cabe señalar que el embarazo es un periodo muy importante para la mujer y que, sumado a la pandemia, un acontecimiento histórico, posiblemente pocas o ninguna situación haya quedado en el olvido.

Los hallazgos de este estudio son de suma relevancia, dados los numerosos cambios impuestos a los servicios de salud durante el período de pandemia, algunos de los cuales se mantuvieron aún después de la misma, como fue el caso de las teleconsultas.

Además, los resultados pueden contribuir a la práctica profesional de quienes asisten a las embarazadas durante la atención prenatal, y mejorar su comprensión acerca de los caminos recorridos en la búsqueda de atención, así como la necesidad de inclusión y aceptación de los familiares y de los aspectos culturales a lo largo de la atención prenatal.

## Conclusión

El itinerario terapéutico seguido por las mujeres embarazadas en búsqueda de atención de salud durante la pandemia de COVID-19 se configuró en tres subsistemas: familiar, profesional y cultural. Las mujeres buscaron primero el apoyo de sus familias, en busca de afecto, cuidados, orientación y apoyo, y luego buscaron el apoyo profesional, que encontraron en la Atención Primaria de Salud, principal puerta de entrada a los cuidados sanitarios, durante todo el período gestacional, parto y puerperio. Muchas mujeres también se acercaron, en el contexto cultural, a las creencias como instrumento de fortalecimiento para el cuidado, lo que ocurrió de forma asociada con los demás subsistemas.

Con la vivencia del embarazo durante la pandemia de COVID-19, los caminos recorridos para buscar atención fueron aún más difíciles para estas gestantes, atravesados por dudas e incertidumbres, además de una nueva configuración de rutinas y cuidados adoptados por los servicios de salud.

Muchas mujeres embarazadas enfrentaron dilemas a la hora de buscar servicios de salud durante la pandemia, por lo que es impostergable garantizar el acceso continuo a la atención prenatal. Se espera que el presente estudio llame a una reflexión sobre la importancia de la atención individualizada y holística a la mujer embarazada, buscando comprender y respetar sus creencias y forma de vida, su comprensión, ansiedades y necesidades momentáneas, a fin de brindar una atención prenatal cualificada, especialmente en situaciones adversas, como la pandemia de COVID-19.
